# Modulation of Macrophage Polarization by Traditional Chinese Medicine in HFpEF: A Review of Mechanisms and Therapeutic Potentials

**DOI:** 10.3390/ph18091317

**Published:** 2025-09-02

**Authors:** Chunqiu Liu, Jinfeng Yuan, Peipei Cheng, Tao Yang, Qian Liu, Tianshu Li, Chuyi Li, Huiyan Qu, Hua Zhou

**Affiliations:** 1 Institute of Cardiovascular Disease of Integrated Traditional Chinese and Western Medicine, Shuguang Hospital Affiliated to Shanghai University of Traditional Chinese Medicine, Shanghai 201203, China; 18703311364@163.com (C.L.);; 2 Branch of National Clinical Research Center for Chinese Medicine Cardiology, Shuguang Hospital Affiliated to Shanghai University of Traditional Chinese Medicine, Shanghai 201203, China; 3 Department of Cardiovascular Disease, Shuguang Anhui Hospital Affiliated to Shanghai University of Traditional Chinese Medicine, Hefei 230031, China

**Keywords:** macrophage, traditional Chinese medicine, heart failure with preserved ejection fraction, mechanisms, signaling pathway

## Abstract

Heart failure with preserved ejection fraction (HFpEF) is a multifactorial cardiovascular disorder characterized by diastolic dysfunction, systemic inflammation, and myocardial fibrosis. Emerging evidence indicates that macrophage polarization imbalance plays a central role in HFpEF pathogenesis. Traditional Chinese medicine (TCM) has demonstrated therapeutic potential in modulating macrophage activity through pathways such as NO/cGMP/PKG, TGF-β/Smads, and PI3K/Akt, thereby exerting anti-inflammatory, antifibrotic, and antioxidant effects. In this review, we conducted a literature search in PubMed, Google Scholar, Web of Science, and CNKI for studies published up to May 2025, using the terms “HFpEF”, “Traditional Chinese Medicine”, and “macrophage”. A total of 19 relevant studies were included. We highlight representative TCM metabolites and TCM formulas, such as resveratrol, Qishen Yiqi Pill, Shenfu Injection, etc. And we summarize their mechanisms in regulating M1/M2 macrophage polarization. Finally, we identify current challenges, including limited HFpEF-specific models and insufficient mechanistic validation, and propose directions for future research.

## 1. Introduction

Heart failure (HF) is the endpoint of many cardiovascular diseases and has been recognized as a global health crisis [1,2]. It is classified based on left ventricular ejection fraction (LVEF) into HF with reduced ejection fraction (HFrEF), HF with preserved ejection fraction (HFpEF), and HF with mildly reduced ejection fraction (HFmrEF) [3]. Notably, HFpEF (LVEF ≥ 50%) constitutes at least half of all HF cases [4,5]. Compared to HFrEF, its prevalence is increasing at a rate of 1% per year [6]. Common comorbidities and risk factors associated with HFpEF include hypertension, coronary artery disease, obesity, metabolic syndrome, renal dysfunction, and arrhythmias. The currently recognized underlying mechanisms of HFpEF are abnormal calcium processing in cardiomyocytes, altered titin phosphorylation status and chronic inflammation leading to cardiac hypertrophy, myocardial fibrosis, and endothelial dysfunction [7,8]. Recent research has highlighted immune dysregulation, particularly involving macrophages, as central to HFpEF development [9]. This has shifted research interest toward immunomodulatory strategies. Due to mechanistic differences, treatments effective for HFrEF are generally ineffective for HFpEF [10]. Sodium–glucose cotransporter 2 inhibitors (SGLT2i) are among the few drug classes with a Class I recommendation in current clinical guidelines for the treatment of HFpEF. Emerging evidences suggest that their therapeutic benefits may be mediated through the modulation of macrophage polarization [11,12].

The immune system, especially macrophages, plays a pivotal role in HFpEF pathogenesis [13]. Macrophages are extensively distributed throughout cardiac tissue and comprise both resident and recruited macrophages [14]. Based on C-C chemokine receptor type 2 (CCR2) expression, cardiac macrophages are categorized into CCR2^−^ embryonically derived resident populations and CCR2^+^ monocyte-derived subsets [15,16]. The CCR2^−^ and a few CCR2^+^ subpopulations constitute cardiac resident macrophages [17]. Through multi-target modulation of inflammatory cascades, fibrotic remodeling, and microvascular endothelial dysfunction, they synergistically preserve cardiac equilibrium and facilitate myocardial tissue regeneration [18,19]. In HFpEF models, single-cell RNA sequencing and flow cytometry have revealed an expansion of pro-inflammatory macrophages in HFpEF hearts [20]. This change in the microenvironment is indicative of cardiac diastolic dysfunction and myocardial fibrosis [20,21].

Macrophage polarization is a critical immunological mechanism influencing HFpEF progression [7,8]. M1 macrophages, activated via TLR4 ligands such as LPS, secrete pro-inflammatory cytokines like IL-6 and TNF-α, aggravating inflammation. Conversely, M2 macrophages, stimulated by IL-4 and IL-13, facilitate fibrotic tissue remodeling. However, excessive M2 activity may contribute to increased myocardial stiffness [22]. The imbalance of macrophage polarization can exacerbate HFpEF [23].

Traditional Chinese medicine (TCM) is increasingly recognized as a source of bioactive compounds capable of immunomodulation. Recent investigations have focused on TCM’s potential to regulate macrophage polarization in cardiovascular diseases such as atherosclerosis and coronary microvascular dysfunction [24,25]. Emerging studies have identified compounds such as berberine, corilagin, and baicalin that directly influence macrophage polarization via pathways like NF-κB, MAPK, and NLRP3 [26]. Several reviews have summarized the role of TCM in regulating macrophage polarization in cardiovascular and inflammatory diseases [26,27]. However, the study of macrophage polarization as a therapeutic target for HFpEF remains in its early exploratory stages. In this review, we focus on the pathophysiology related to macrophages of HFpEF and the signaling pathways linking TCM to macrophage regulation. By integrating evidence from both preclinical and clinical studies, we also evaluate the translational potential and limitations of current findings and provide a framework for future targeted therapeutic strategies.

## 2. Materials and Methods

### 2.1. Search Method

**Figure 1:**
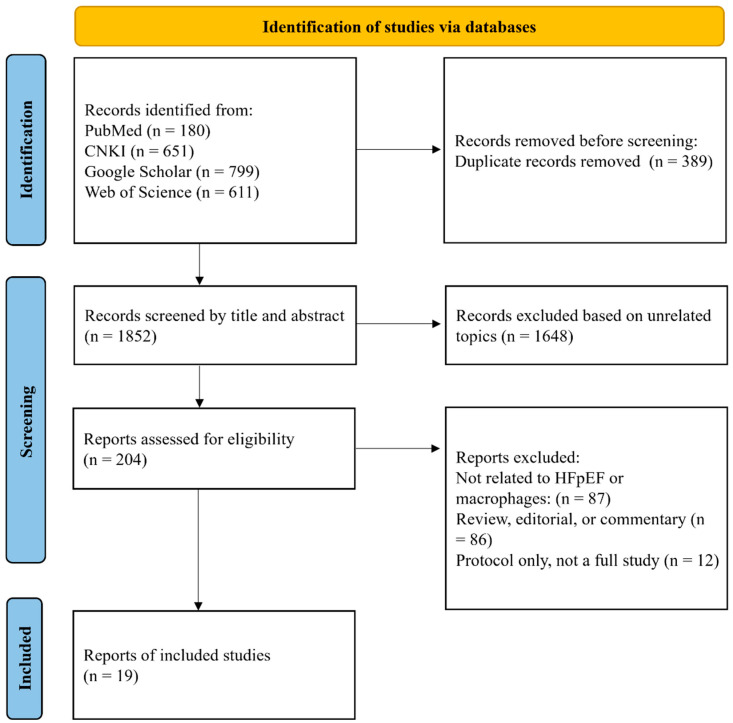
Study selection flowchart.

Relevant literature was retrieved from several scientific databases, including PubMed (https://pubmed.ncbi.nlm.nih.gov/), accessed on 25 May 2025; China National Knowledge Infrastructure (CNKI) (https://www.cnki.net), accessed on 25 May 2025; Google Scholar (https://scholar.google.com.hk/?hl=zh-CN), accessed on 25 May 2025; Web of Science (https://webofscience.clarivate.cn/wos/alldb/basic-search), accessed on 25 May 2025. The search encompassed all records from the inception of each database to May 2025. A combination of Medical Subject Heading (MeSH) terms and free-text keywords such as “macrophage”, “HFpEF”, “Traditional Chinese medicine”, “herb”, and related terminologies were employed to ensure comprehensive retrieval (Figure 1).

### 2.2. Inclusion and Exclusion Criteria

The retrieved literature must be the original animal, cellular, or clinical research and meet the following criteria: (1) detected relevant indicators of macrophages, (2) treated with TCM formula or active metabolites of TCM, and (3) LVEF ≥ 50% or no evidence indicating LVEF < 50%, along with evidence of structural or functional cardiac abnormalities associated with left ventricular diastolic dysfunction/elevated diastolic pressure. Examples include ventricular dilation, myocardial hypertrophy, myocardial fibrosis, elevated BNP levels, etc.

The exclusion criteria comprised studies lacking ethical approval, incomplete datasets compromising methodological rigor, retracted publications due to scientific validity concerns, and other types of studies (such as reviews, meta-analyses, etc.).

## 3. Dual Role of Macrophage Phenotypic Plasticity in the Pathogenesis of HFpEF

Macrophages have traditionally been classified into pro-inflammatory M1 and anti-inflammatory M2 phenotypes [28]. However, studies have revealed a spectrum of intermediate macrophage phenotypes, including Mox, Mhem, M4, and M(Hb), each exhibiting distinct functional properties [17]. ApoE−/− mice fed a Western diet exhibit key HFpEF-like features, including impaired diastolic function, reduced exercise tolerance, and increased pulmonary congestion linked to cardiac lipid overload and reduced polyunsaturated fatty acids. Single-cell RNA sequencing of CD45^+^ cardiac cells from this model highlighted the impact of metabolic stress on cardiac immune cells, particularly macrophages [29]. Further analyses indicated that HFpEF is associated with the infiltration of CCR2^+^ resident cardiac macrophages expressing Spp1 [30], as well as an increase in pro-inflammatory macrophage subsets in murine HFpEF models [20]. In this review, we focus primarily on the classical M1 and M2 phenotypes while acknowledging the broader diversity of macrophage subsets and their context-dependent roles in HFpEF.

**Figure 2:**
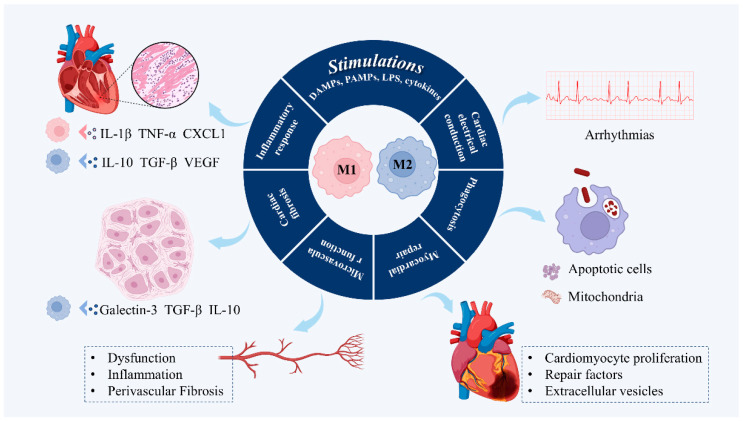
Roles of macrophages in HFpEF. DAMPs: damage-associated molecular patterns; PAMPs: pathogen-associated molecular patterns; LPS: lipopolysaccharide; IL-1β: interleukin-1 beta; TNF-α: tumor necrosis factor alpha; CXCL1: C-X-C motif chemokine ligand 1; TGF-β: transforming growth factor beta; VEGF: vascular endothelial growth factor.

Macrophages in HFpEF should be understood as a dynamic continuum rather than a binary M1/M2 classification. M1 macrophages promote innate immune responses and damage-associated molecular pattern (DAMP) clearance through pro-inflammatory cytokine release, but chronic activation drives cardiomyocyte apoptosis and endothelial dysfunction. M2 macrophages support repair by modulating extracellular matrix remodeling and stimulating angiogenesis, yet excessive M2 activity contributes to fibrosis and myocardial stiffening (Figure 2). Resident CCR2^−^ macrophages and other ontogenically distinct subsets are essential for homeostasis, while infiltrating CCR2^+^ populations drive maladaptive remodeling [31]. Single-cell studies have further identified HFpEF-specific macrophage states shaped by metabolic and hemodynamic stress [29]. Together, these findings highlight that therapeutic strategies should aim not at simply “pushing” macrophages toward M2 polarization but at maintaining a balanced spectrum of macrophage phenotypes to prevent the transition from adaptive to maladaptive remodeling in HFpEF.

### 3.1. Regulating Inflammatory Response

Biomarker analyses in HF patients have revealed distinct pathogenic differences between HFpEF and HFrEF [32]. In HFpEF, inflammation and fibrosis are more prominent than in HFrEF. Notably, during clinical deterioration, systemic levels of inflammatory cytokines, such as serum tumor necrosis factor-α (TNF-α) and interleukin-6 (IL-6), are elevated by approximately 1.3- to 2.4-fold compared to levels observed in stable HFpEF [32]. These findings suggest a strong association between clinical worsening in HFpEF and heightened inflammatory responses [33].

As an essential component of the immune system, macrophages play a key role in the initiation, progression, and recovery of the inflammatory response following cardiac tissue injury [34]. In the normal state, the heart is dominated by cardiac resident macrophages [35]. After an injury occurs, the heart enters an acute inflammatory phase immediately [36]. Resident macrophages are drastically reduced, and circulating monocytes are recruited to the injured heart. These monocytes localize and differentiate into CCR2^+^ macrophages, which further polarize into pro-inflammatory and anti-inflammatory phenotypes [37,38].

In the early stages of inflammation, macrophages exhibit a pro-inflammatory M1 phenotype to initiate an immune response and clear cellular debris. M1 macrophage is characterized by the secretion of pro-inflammatory cytokines, growth factors, and chemokines such as IL-1β, TNF-α, C-X-C motif ligand (CXCL) 1, and matrix metalloproteinases (MMPs) [39,40]. However, the prolonged presence of M1 macrophages hinders the regression of scar formation. Over time, the myocardium enters a proliferative and reparative phase, when macrophages exhibit an M2 anti-inflammatory phenotype to inhibit excessive inflammation, promote myocardial repair, and produce a variety of anti-inflammatory cytokines and pro-angiogenic and pro-repair factors, such as IL-10, transforming growth factor-β (TGF-β), and vascular endothelial growth factor (VEGF) [41,42]. Single-cell sequencing further confirmed the connection between macrophages and cardiomyocytes and revealed that various inflammatory cytokines secreted by macrophages affect the hypertrophy, fibrosis, and autophagy of myocardial cells during the progression of HFpEF [29].

### 3.2. Regulation of Cardiac Fibrosis

Myocardial fibrosis represents a hallmark pathological feature of HFpEF, reflecting a maladaptive remodeling process that contributes critically to disease progression [20]. While moderate fibrotic responses may serve a reparative function in maintaining structural integrity after myocardial injury, excessive and persistent fibrosis compromises ventricular compliance and diastolic filling, ultimately impairing cardiac function.

At the cellular level, cardiac fibroblasts are central orchestrators of fibrotic remodeling, constituting approximately 60–70% of non-myocyte cardiac cells [43]. Upon pathological stimulation, these fibroblasts undergo phenotypic transformation into myofibroblasts [44]. Myofibroblasts are a secretory cell type characterized by upregulated expression of α-smooth muscle actin and robust secretion of extracellular matrix (ECM) components such as collagen types I and III [43]. This transformation marks a critical step in the establishment of a pro-fibrotic microenvironment.

The deposition of ECM is tightly modulated by immune cell populations, especially M2 macrophages [45]. While M2 macrophages contribute to inflammation resolution and early tissue repair, they also secrete mediators with pro-fibrotic potential. Among these, galectin-3 has emerged as a key molecular mediator and early biomarker of fibrotic remodeling. Secreted by M2 macrophages, galectin-3 activates both fibroblasts and macrophages in a feed-forward loop, exacerbating fibrotic progression [15,46]. In parallel, M2 macrophages secrete profibrotic cytokines such as TGF-β [47] and IL-10 [48]. Furthermore, macrophages themselves can undergo phenotypic conversion into myofibroblast-like cells, a process termed macrophage-to-myofibroblast transition (MMT), further amplifying the fibrotic burden [47]. These findings emphasize that M2 macrophages can support repair when properly regulated but contribute to maladaptive remodeling if they are excessively or persistently activated. Mass spectrometry and flow cytometry confirm M2 macrophage infiltration and activity after ischemia–reperfusion (IR) injury [49]. Targeting these pathways holds substantial therapeutic promise. For instance, blocking IL-1β signaling with monoclonal antibodies has been shown to attenuate myocardial fibrosis and improve ventricular compliance and overall cardiac function [50].

### 3.3. Regulation of Microvascular Function

Low-grade chronic systemic inflammation is a central upstream driver of coronary microvascular dysfunction in HFpEF, serving as a pathophysiological bridge between systemic comorbidities and cardiac remodeling [51]. Unlike the overt ischemic injury commonly seen in HFrEF, HFpEF is characterized by insidious microvascular injury that gradually impairs myocardial structure and function. The resulting endothelial dysfunction impairs vasodilation, reduces perfusion reserve, and fosters a pro-fibrotic microenvironment, contributing to hallmark features of HFpEF, including increased interstitial and perivascular fibrosis [52,53]. Perivascular fibrosis increased the diffusion distance of oxygen and nutrients [54]. This vascular dysfunction triggers macrophage infiltration, disrupts paracrine signaling, and reduces nitric oxide availability.

Microvascular pathology is not only more prevalent in HFpEF compared to HFrEF but also appears more tightly coupled to extracardiac comorbidities [55]. Common systemic conditions such as obesity, type 2 diabetes, hypertension, and chronic kidney disease impose persistent inflammatory stress on the endothelium. This in turn disrupts the coronary microvascular barrier, promoting oxidative stress and endothelial-to-mesenchymal transition, which accelerates fibrotic remodeling [13]. Endothelial dysfunction appears in the early stages of HFpEF [56]. Increased expression of endothelial adhesion molecules causes monocytes to be recruited to the heart. Left ventricular biopsy shows increased VCAM expression in patients with HFpEF [57]. In patients with HFpEF, echocardiographic indicators of diastolic dysfunction (E/e’, LVDD) are associated with monocyte recruitment, especially M2 macrophages [58,59].

### 3.4. Promotes Myocardial Repair

Cardiomyocytes are the cell type that makes up the myocardium, accounting for about one-third of the myocardium, with an average of 1 cardiomyocyte surrounded by every five macrophages [60]. Cardiomyocyte proliferation is the main driver of cardiac regeneration, and adult mammals progressively lose the ability to proliferate cardiomyocytes, which makes it difficult to fully recover if myocardial injury occurs [61].

Following myocardial infarction (MI), HFpEF can emerge, during which M2 macrophages play a pivotal role in cardiac recovery. One of their key mediators, IL-10, has been shown to facilitate myocardial repair by modulating inflammation and promoting tissue regeneration [62]. In addition to cytokine release, M2 macrophages also contribute to cardiac repair through the secretion of extracellular vesicles (EVs), which have emerged as vectors for delivering reparative signals [63].

FOS and ALOX5 are genes associated with visceral adipocytes and are highly expressed in macrophages under hypoxic conditions. Single-cell sequencing revealed that these genes are significantly upregulated in the subepicardial tissue of post-MI HFpEF patients. These findings suggest that FOS and ALOX5 may modulate macrophage response to hypoxia, thereby promoting cellular senescence and the progression of post-MI HFpEF, while inhibition of these genes could potentially enhance cellular repair and mitigate HFpEF progression [64].

### 3.5. Phagocytosis

Macrophage-mediated efferocytosis, specifically the clearance of necrotic cells and mitochondrial debris, serves as a gatekeeper against inflammatory amplification and fibrotic cascades in cardiac pathology [65]. When this phagocytic process is impaired, unresolved cellular debris triggers chronic immune activation, disrupting tissue homeostasis and promoting maladaptive cardiac remodeling [66]. Key failure points include the apoptosis of resident cardiac macrophages, inactivation of the MER tyrosine kinase (MerTK) receptor, and overexpression of “don’t eat me” signals such as CD47, all of which block efficient phagocytosis [67].

Myocardial mitochondrial dysfunction is present in the course of HFpEF [68]. Normally, cardiomyocytes internalize macrophage-derived mitochondria via a lattice clathrin-dependent and/or lipid raft-mediated endocytosis pathway [69]. The resulting cardiomyocytes establish a complex mitochondrial network that maintains cardiac output and provides adequate bioenergetic support [70]. When mitochondria are dysregulated, cardiomyocytes release mitochondria via autophagocytes, which are recognized and phagocytosed by cardiac resident macrophages with the help of the receptor Mertk, thus preventing inflammatory vesicle activation and autophagy blockade and ultimately maintaining cardiac homeostasis [71].

This dynamic mitochondrial exchange is not merely a metabolic adaptation but a crucial immunoregulatory mechanism. Disruption of this axis—for instance, through SIRT3 deficiency—leads to MerTK inhibition, mitochondrial iron overload, and upregulation of proinflammatory cytokines in cardiac macrophages, particularly under angiotensin II (Ang-II) stress [72]. Recent evidence further underscores the role of mitochondria in regulating macrophage phenotype and function. Mitochondrial signals modulate macrophage polarization and efferocytosis capacity, directly influencing the balance between tissue repair and inflammation [73]. Thus, mitochondrial integrity and macrophage phagocytic competence are tightly interlinked, forming a homeostatic circuit essential for suppressing inflammation and preserving myocardial structure.

### 3.6. Facilitating Cardiac Electrical Conduction

Cardiac macrophages are not only immune regulators but also active participants in myocardial electrophysiology. They are widely distributed across the sinoatrial and atrioventricular nodes, as well as the atria and ventricles, where they influence electrical conduction [15,71]. Normal electrical conduction depends on gap junction-mediated current transfer via connexin complexes [19,74]. Resident macrophages establish direct electrical coupling with myocardial cells via connexin 43, leading to resting membrane depolarization, early action potential shortening, and late action potential prolongation [15,75,76].

A common arrhythmic comorbidity of HFpEF is atrial fibrillation [77]. Although there are well-established catheter ablation techniques for the treatment of AF, a recent meta-analysis found that catheter ablation did not significantly benefit patients with HFpEF [78], while macrophages may be a new therapeutic entry point. Emerging evidence implicates macrophages—particularly pro-inflammatory subsets—as active contributors to atrial arrhythmogenesis. Monocyte-derived macrophages can undergo phenotypic shifts that favor a pro-inflammatory state, thereby promoting atrial electrical instability [79]. These findings underscore the causal role of immune cell-driven microenvironmental remodeling in atrial arrhythmia.

Changes in macrophage subtypes and numbers are associated with ventricular arrhythmias [80]. Moreover, depletion of M1 macrophages suppresses excessive activation of the left stellate ganglion, reducing MI-induced malignant arrhythmias [81]. In the dTGR model of HFpEF, there is a massive infiltration of macrophages accompanied by remodeling of gap junctions [82]. This disrupts electrical coupling between cardiomyocytes and increases the risk of ventricular arrhythmias.

These observations suggest that the role of macrophages in arrhythmogenesis depends on subtype balance, activation status, and distribution within the myocardium. Rather than acting uniformly as pro- or anti-arrhythmic agents, macrophages appear to function along a spectrum of regulatory states. Future research should aim to decipher how imbalances in macrophage phenotype, particularly pro-inflammatory biases or excessive infiltration, contribute to electrical instability in both atrial and ventricular settings. Such understanding may offer new immunomodulatory strategies to prevent arrhythmias in HFpEF and related cardiac pathologies.

## 4. Signaling Pathways Driving Macrophage Polarization

**Figure 3:**
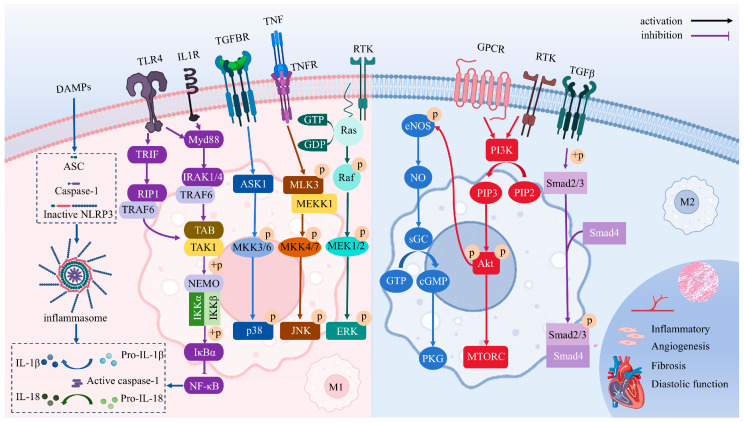
The signaling pathway involved in regulating macrophage polarization in HFpEF. ASC: apoptosis-associated speck-like protein containing a CARD; NLRP3: NOD-like receptor family pyrin domain-containing 3; TLR4: Toll-like receptor 4; TRIF: TIR domain-containing adapter-inducing interferon-β; RIP1: receptor-interacting protein 1; TRAF6: TNF receptor-associated factor 6; IL-1R: interleukin-1 receptor; Myd88: myeloid differentiation primary response 88; IRAK1/4: interleukin-1 receptor-associated kinase 1/4; TAK1: transforming growth factor-beta-activated kinase 1; TAB: TAK1-binding protein; IκBα: inhibitor of nuclear factor kappa-B alpha; IKK: IκB kinase; NF-κB: nuclear factor kappa-light-chain-enhancer of activated B cells; NEMO: NF-κB essential modulator; TGFBα: transforming growth factor beta alpha; ASK1: apoptosis signal-regulating kinase 1; MKK3/6: mitogen-activated protein kinase kinase 3/6; TNFR: tumor necrosis factor receptor; MLK: mixed-lineage kinase; MEKK1: mitogen-activated protein kinase 1; JNK: c-Jun N-terminal kinase; RTK: receptor tyrosine kinase; Ras: rat sarcoma virus protein; Raf: rapidly accelerated fibrosarcoma; ERK: extracellular signal-regulated kinase; eNOS: endothelial nitric oxide synthase; NO: nitric oxide; sGC: soluble guanylate cyclase; GTP: guanosine triphosphate; cGMP: cyclic guanosine monophosphate; PKG: protein kinase G; GPCR: G protein-coupled receptor; PI3K: phosphoinositide 3-kinase; PIP3: phosphatidylinositol (3,4,5)-trisphosphate; Akt: protein kinase B; MTORC: mechanistic target of rapamycin complex.

Multiple signaling pathways have been implicated in regulating macrophage polarization and contributing to disease progression in HFpEF. Key pathways, including NO/cGMP/PKG, TGF-β/Smads, TLRs/NF-κB, PI3K/AKT, NLRP3 inflammasome, and MAPK, are known to mediate macrophage polarization. In HFpEF models, dysregulation of these pathways contributes to persistent low-grade inflammation, endothelial dysfunction, and ventricular remodeling. Clarifying these pathways helps identify how macrophage imbalance contributes to HFpEF and guides potential interventions (Figure 3).

### 4.1. NO/cGMP/PKG

NO is released by endothelial nitric oxide synthase (eNOS), neuronal nitric oxide synthase (nNOS), and inducible nitric oxide synthase (iNOS). Under physiological conditions, NO is primarily released by eNOS, triggering a cascade of cellular signaling reactions. NO stimulates soluble guanylate cyclase (sGC), which catalyzes the conversion of guanosine triphosphate (GTP) into cyclic guanosine monophosphate (cGMP). This activation triggers protein kinase G (PKG), a key mediator of NO/cGMP-dependent vasodilation in vascular smooth muscle cells, while concurrently exerting cardioprotective effects through anti-hypertrophic, anti-fibrotic, and pro-angiogenic actions in the myocardium [9,83].

The NO/cGMP/PKG signaling pathway is recognized as a potential mechanism contributing to HFpEF. In HFpEF, microvascular endothelial inflammation reduces NO bioavailability, impairing the NO/cGMP/PKG pathway and thereby promoting structural and functional abnormalities in the cardiac and coronary microvasculature [84,85]. SGLT2 inhibitors, recommended by clinical guidelines for HFpEF, have been reported to reverse left ventricular concentric remodeling, potentially via activation of the NO/cGMP/PKG pathway and facilitation of macrophage polarization toward M2 phenotypes [86,87]. In addition, cGMP/PKG signaling may inhibit M1 macrophage polarization [88]. More recently, preliminary evidence from multi-omics and network pharmacology analyses has suggested that QiShenYiQi dripping pills and ZWT might improve cardiac inflammation and fibrosis in HFpEF by modulating the cGMP/PKG signaling pathway [89,90]. In further animal experiments, an HFpEF model was established using a combination of a high-fat diet and Nω-nitro-L-arginine methyl ester (L-NAME) in drinking water. The cardioprotective effects of QSYQ in HFpEF mice were attenuated after myocardial PKG knockdown mediated by RNA interference [90].

On the other hand, under pathological conditions, M1 macrophage polarization induced by interferon-gamma (IFN-γ) may be associated with NO production mediated by iNOS [91]. Excessive NO generated by iNOS can S-nitrosylate and activate HIF-1α, triggering glycolysis and pro-inflammatory programs in M1 macrophages [92]. A study has found that iNOS knockout reduces Akt S-nitrosylation, thereby alleviating oxidative stress in HFpEF mice [93].

### 4.2. TGF-β/Smads

TGF-β, particularly TGF-β1, is associated with extracellular matrix remodeling and is a well-known pro-fibrotic cytokine [94]. It can be secreted by M2 macrophages and also exerts anti-inflammatory and anti-atherosclerotic effects [95]. It can induce fibroblasts to synthesize ECM and remodel tissue. While TGF-β is broadly implicated in cardiac fibrosis, its role in HFpEF appears more distinct due to the disease’s unique pathophysiology [15,96]. Unlike the replacement fibrosis seen in HFrEF, TGF-β in HFpEF induces perivascular and interstitial fibrosis through fibroblast activation and excessive ECM deposition, leading to ventricular stiffening and impaired diastolic relaxation [97].

The TGF-β/Smad3 signaling pathway is a key mediator of ventricular fibrosis, hypertrophy, and dysfunction [98]. TGF-β mediates its biological effects through binding to TGFβRI/TGFβRII receptors, initiating Smad2/3 phosphorylation. The phosphorylated Smad2/3 then complexes with Smad4 and translocates into the nucleus, where it upregulates genes associated with fibroblast activation, collagen synthesis, and pathological extracellular matrix accumulation [99,100]. Inflammation indirectly modulates TGF-β via macrophage phagocytic activity and is amplified by cardiac fibroblast activation in response to mature collagen deposition [101]. Activation of p-Smad2/Smad3 can promote an increase in M2 macrophages and have anti-inflammatory and restorative effects in the early stages of injury [102].

While the TGF-β cascade may confer initial cardioprotection through induction of fibroblast ECM phenotypes and suppression of pro-inflammatory ECM fragment formation, its chronic overactivation in pressure-overloaded cardiomyocytes and fibroblasts ultimately drives cardiac fibrosis and functional impairment [103]. Deletion of TGF-β receptors and Smad2/3 has been shown to suppress fibrosis and ECM remodeling gene programs [104]. In an animal study, analysis of protein and gene expression levels in HFpEF model rats revealed that the TGF-β1/Smads signaling pathway was excessively activated in the model group. Treatment with sacubitril/valsartan effectively suppressed the activation of this pathway [105].

### 4.3. TLRs/NF-κB

Pattern recognition receptors (PRRs) mediate essential pathogen detection in innate immunity [106]. The PRR family consists of five types of receptors, among which Toll-like receptors (TLRs) have been the most extensively studied in mammals [107]. Among the 10 TLRs identified in humans, TLR4 exhibits the highest expression in the heart [108,109]. Upon recognizing LPS, TLR4 activates two major adaptor protein pathways: the myeloid differentiation primary response 88 (MyD88)-dependent and TIR domain-containing adapter-inducing interferon-β (TRIF)-dependent signaling cascades [110]. These pathways converge on nuclear factor kappa-light-chain-enhancer of activated B cells (NF-κB) activation through a series of molecular events involving IL-1 receptor-associated kinases (IRAKs), TNF receptor-associated factor 6 (TRAF6), and receptor-interacting protein 1 (RIP1) [111,112]. This leads to inhibition of nuclear factor κ-B (IκB) degradation and subsequent NF-κB translocation into the nucleus, where it drives pro-inflammatory gene expression in macrophages [112].

As the principal transcriptional effector of TLR4 signaling, NF-κB orchestrates the pro-inflammatory gene program in M1 macrophages, directly regulating key mediators including TNF-α, IL-1β, IL-6, IL-12, and cyclooxygenase-2 [113]. Prolonged maladaptive TLR4 signaling and excessive NF-κB activation lead to sustained inflammatory cytokine release by cardiac macrophages, activating chronic inflammation and ultimately resulting in cardiac remodeling and functional deterioration [106]. Preventing NF-κB phosphorylation attenuates oxidative stress and inflammatory responses in HFpEF mice [114]. Qiliqiangxin, a TCM formula, has demonstrated efficacy in modulating this pathway [115]. In HFpEF rats, Qiliqiangxin treatment downregulated myocardial NF-κB expression, reduced M1-associated cytokine levels, and enhanced IL-10 production. IL-10 is a marker of M2 macrophage activity. These findings suggest that its cardioprotective effect may be mediated, at least in part, by restoring the M1/M2 balance via suppression of maladaptive NF-κB activation.

### 4.4. NLRP3 Inflammasome

The NOD-like receptor family pyrin domain-containing 3 (NLRP3) inflammasome is a protein complex in macrophages that detects danger signals inside cells [116]. It includes NLRP3 (the sensor), ASC (the adaptor), and caspase-1 (the effector) [11,117]. Its activation has two steps: initiation and activation. First, signals like TLRs or IL-1R activate NF-κB, which increases the levels of NLRP3, pro-IL-1β, and pro-IL-18. Then, cell stress or DAMPs trigger NLRP3 to recruit apoptosis-associated speck-like protein containing a CARD (ASC), which binds pro-caspase-1. This forms the inflammasome and activates caspase-1 [118]. Caspase-1 then processes pro-IL-1β and pro-IL-18 into their active forms, which are released to promote inflammation [119].

NLRP3 activation is increasingly recognized as a driving factor for M1 macrophage polarization. The evidence in mice suggests that when NLRP3-Caspase1/IL-1β is inhibited, the levels of IL-10 anti-inflammatory cytokines increase in the ischemic myocardium and M1 macrophages [120]. Both IL-1β and IL-18 are pro-inflammatory factors released by M1 macrophages that can worsen myocardial injury. In clinical studies, IL-1 blockade with anakinra significantly improved ventilatory efficiency (V_E_/Vco_2_ slope) in patients with HFpEF and reduced high-sensitivity CRP levels, suppressing systemic inflammatory responses [121,122,123]. In terms of active metabolites of TCM research, there have been more abundant studies on colchicine for the treatment of cardiovascular diseases [124]. A study reported that colchicine improves cardiac diastolic function and fibrosis in high-salt diet-induced HFpEF rats by inhibiting the NLRP3 inflammasome pathway [125].

### 4.5. PI3K/Akt

Phosphatidylinositol 3-kinase (PI3K) is activated by receptor tyrosine kinase (RTK) or G protein-coupled receptor (GPCR). It can convert phosphatidylinositol-4,5-bisphosphate (PIP2) to phosphatidylinositol-3,4,5-trisphosphate (PIP3) [126]. PIP3 then recruits and activates protein kinase B (Akt) through phosphorylation by phosphoinositide-dependent kinase 1 (PDK1) and mammalian Target of Rapamycin Complex 2 (mTORC2) [22,127]. Akt isoforms regulate macrophage polarization: Akt1 loss promotes M1, while Akt2 loss favors M2 [22]. The PI3K/Akt signaling pathway supports macrophage-mediated tissue repair, enhances angiogenesis, and alleviates fibrosis in the heart [26,128]. It can promote macrophage phenotypic switching from M1 to M2 [129,130].

Emerging studies suggest that impaired PI3K/Akt signaling may underlie defective macrophage-mediated cardiac repair in HFpEF. The PI3K/Akt signaling pathway supports macrophages in repairing damaged cardiac tissue, promoting angiogenesis, and reducing fibrosis [126,131]. Furthermore, excessive iNOS-derived NO production by macrophages may induce Akt S-nitrosylation in the HFpEF murine model [93]. Attenuation of Akt activity can disrupt downstream signaling cascades, such as the NO/cGMP/PKG pathway. This crosstalk may further exacerbate endothelial dysfunction and diastolic impairment.

### 4.6. MAPK

Mitogen-activated protein kinase (MAPK) signaling, including extracellular signal-regulated kinase (ERK), c-Jun N-terminal kinase (JNK), and p38 pathways [26,132,133], plays a central role in regulating macrophage polarization and is increasingly implicated in the pathogenesis of HFpEF. While the canonical MAPK cascades coordinate cellular responses to stress and inflammation, their specific activation patterns in HFpEF have only recently gained attention. In HFpEF models, activation of ERK1/2 and JNK is associated with enhanced M1-like macrophage polarization, particularly in response to elevated circulating leucine-rich α-2 glycoprotein 1 (LRG1) levels [134]. Moreover, sacubitril/valsartan has been shown to suppress M1 polarization via the NPR-C/cAMP/JNK/c-Jun axis [135]. This indicates that targeting MAPK signaling may help resolve inflammation in HFpEF.

Similarly, p38 MAPK, a key pro-inflammatory mediator, is activated by stress stimuli [26]. And it contributes to myocardial remodeling in HF [136]. Studies have demonstrated that inhibition of p38 promotes the M1-to-M2 switch, reducing cardiac fibrosis and inflammation [137,138]. Notably, phosphorylation of MAPKs is upregulated in salt-sensitive hypertensive HFpEF models, and blockade of this pathway mitigates myocardial hypertrophy and fibrosis [139,140]. These findings highlight MAPK signaling not only as a driver of macrophage-mediated inflammation but also as a potential therapeutic target in HFpEF.

## 5. TCM Treats HFpEF by Modulating Macrophages

### 5.1. Active Metabolites of TCM

Investigations of active metabolites derived from TCM have provided compelling evidence for their therapeutic potential in HFpEF. These isolated active metabolites facilitate a targeted and mechanistically elucidated intervention, simplifying the pharmacological complexity inherent in whole TCM formulations. By focusing on specific molecular targets and signaling pathways, these metabolites advance precision medicine approaches for multifactorial cardiovascular pathology. Many active metabolites, such as leonurine, gentiopicroside, and resveratrol, have been demonstrated to have anti-inflammatory, antifibrotic, and anti-oxidative properties, which may help alleviate HFpEF pathophysiology by modulating macrophage polarization (Table 1).

#### 5.1.1. Anti-Inflammatory Activity

Schisandrin B (SchB), a lignan from *Schisandra chinensis*, exerts cardioprotective effects [154]. A study used AngII to induce cardiac remodeling in mice. The model mice exhibited an LVEF ≥ 50%, along with increased left ventricular internal dimension in diastole (LVIDd) and enlarged left ventricular end-diastolic volume (LVEDV). Results showed that SchB had a significant inhibitory effect on macrophages (F4/80) after 2 weeks compared to hydralazine. Comparison with MyD88 knockdown mice suggested that the mechanism involves the MyD88/TLR pathway. In MyD88-deficient cells, AngII-induced inflammation and gene expression were markedly reduced, confirming MyD88 as a key target of SchB [142]. Recent advancements in nanotechnology have enabled macrophage-targeted delivery of SchB, enhancing its stability, circulation time, and therapeutic precision [155,156].

Leonurine, a bioactive alkaloid constituent derived from the TCM *Leonurus japonicus* Houtt. It can improve atherosclerosis by diminishing macrophage lipid accumulation via METTL3-mediated AKT1S1 mRNA stability modulation [157]. In Ang II-induced hypertensive HF in mice (Increased ventricular mass, increased ventricular wall thickness during diastole, enlarged inner diameter, LVEF ≥ 50%), leonurine showed significant cardioprotective effects by attenuating cardiac hypertrophy, fibrosis, and inflammation compared to the model group [145]. The mechanism involves inhibition of MAPK and NF-κB signaling, which reduces macrophage (F4/80) infiltration and cardiomyocyte injury. In vitro application of neonatal rat primary cardiomyocytes further confirmed the above intervention mechanism.

Arctigenin (AG) is a lignan with diuretic, anti-inflammatory, and detoxifying properties. Previous research has found that AG may ameliorate inflammatory diseases by inhibiting PI3K and polarizing M1 macrophages to M2-like macrophages [158]. A study induced MI by ligating the left anterior descending coronary artery and treated with AG for 18 weeks [152]. Ultrasound results showed that all mice had ejection fractions ≥ 50%. AG reduced cardiac injury by suppressing inflammatory macrophages/monocytes and their proinflammatory cytokines. Transcriptomic analysis confirmed that AG regulates macrophage polarization via the NFAT5 pathway. Overexpression of NFAT5 reversed the effects of AG, confirming it as a key target. In vitro experiments using RAW264.7 macrophages and mouse cardiomyocytes further confirmed AG’s anti-inflammatory effects, modulating M1 (CD86) and M2 (CD206) polarization, and demonstrated that this action is mediated via NEAT5.

*Lignum dalbergiae odoriferae*, obtained from the dried heartwood or root of *Dalbergia odorifera* T. Chen, a leguminous plant of the Papilionaceae family. It is traditionally used in TCM for its properties in promoting blood circulation, regulating qi, and alleviating pain. And it has been used to treat cardiovascular diseases [159,160]. Latifolin is a flavonoid derived from *lignum dalbergiae odoriferae*. A study by Zhang [151] showed that Latifolin protected against DOX-induced cardiac dysfunction by inhibiting inflammation-related mechanisms, such as iNOS. In this study, the model exhibited LVEF ≥ 50% and was accompanied by increased end-diastolic ventricular diameter and myocardial fibrosis, closely resembling HFpEF. The result of PCR showed that latifolin decreased the gene expression of M1 markers (iNOS and CD86) and increased the genetic expression of M2 markers (CD206, IL-10, and IL-4R). In peritoneal macrophages, it suppressed inflammatory cytokine production and reduced the ratio of M1/M2.

#### 5.1.2. Anti-Oxidative Stress

Vanillic acid (VA), a plant-derived phenolic metabolite found in *Salvia miltiorrhiza*, mint, *Pueraria lobata*, and related plants, exerts a range of pharmacological activities, including antioxidant, anti-inflammatory, hepatoprotective, and anti-angiogenic effects [161]. One study investigated the effects of VA on ISO-induced cardiac fibrosis in mice (LVEF ≥ 50%; increased ventricular wall thickness, inner diameter, and volume; decreased maximum rate of increase/decrease in left ventricular pressure) and its mechanism of action compared to Drp1 inhibitor [143]. Male C57BL/6J mice were used in the experiment, and after continuous intravenous administration for 14 days, indicators of inflammation and oxidative stress were measured. Flow cytometry results showed that VA decreased the proportion of M1-type macrophages (CD86), elevated the proportion of M2-type macrophages (CD206), and promoted anti-inflammatory responses. In addition, VA increased the activities of antioxidant enzymes such as CAT, GSH, and SOD in cardiac tissues. The above efficacy may be realized through Drp1/HK1/NLRP3.

Resveratrol (RSV) is a natural polyphenol mainly found in grapes and berries. It has a variety of effects, including anti-inflammatory, anti-oxidative stress, and anti-fibrosis [149,162]. A study in which ISO was administered to mice resulted in cardiac dysfunction as evidenced by cardiac hypertrophy and cardiomyocyte fibrosis [148]. After 1 week of RSV treatment, M1 macrophages (CD68) were reduced, and inflammation decreased. The experiment in RAW264 considered that RSV may act through the VEGFB/AMPK/NF-кB pathway. In another study, HFpEF was modeled by unilateral nephrectomy combined with aldosterone infusion [149]. Four weeks after intravenous injection of RSV, mRNA expression of M1-type markers (iNOS, CD86, CD80) decreased in the hearts of mice, while mRNA expression of M2-type markers (Arg1, CD163, CD206) increased. Moreover, RSV significantly reduced HFpEF-induced Smad3 acetylation and inhibited Smad3 transcriptional activity by activating Sirt1. The experimental results of cardiac fibroblasts were consistent with the above. These pathways are central to immune balance and oxidative regulation in HFpEF. Importantly, emerging evidence suggested that RSV may be effectively delivered via macrophage-derived exosomes, offering targeted therapy [163]. This aligns with the need for precise immune modulation in HFpEF and opens new directions for RSV-based interventions.

Cardamonin (CAR) is a flavonoid from *Alpinia katsumadai*. It combats oxidative stress by lowering ROS and MDA levels. In a doxorubicin-induced cardiotoxicity model, CAR activated the Nrf2 pathway, improving heart function and reducing damage after 4 weeks [150]. In this study, the model showed a certain degree of correlation with HFpEF, with LVEF ≥ 50% and cardiac enlargement. F4/80, as a marker of macrophages and inflammatory response, decreased after treatment. Nrf2 is critical in defending against oxidative injury. By enhancing the cell’s antioxidant defenses, CAR may protect the heart from chemotherapeutic damage and inflammation. Moreover, CAR promoted Nrf2-ARE–regulated cytoprotective genes in HL-1 cells in an Nrf2-dependent manner.

#### 5.1.3. Anti-Myocardial Fibrosis

20(S)-ginsenoside Rh2 (20(S)-Rh2), a bioactive metabolite derived from *ginseng*, is known for its qi-enhancing properties and has been widely used in the treatment of immune-related disorders [164,165]. In addition, 20(S)-Rh2 exerts cardioprotective effects by attenuating oxidative stress and lowering myocardial enzyme levels [144]. In a mouse model of hypertensive HF (LVEF ≥ 50% and elevated ANP), 20(S)-Rh2 inhibited myocardial fibrosis, hypertrophy, and macrophage (F4/80) infiltration induced by Ang-II without affecting blood pressure after 2 weeks. As shown by RNA sequencing data, this cardioprotective effect is related to the inhibition of the JNK/AP-1 signaling pathway. In neonatal rat ventricular myocytes (NRVMs), 20(S)-Rh2 lost its anti-inflammatory effect when JNK or AP-1 was knocked out. The dependence of 20(S)-Rh2 on this pathway suggests a highly targeted and manipulative mechanism, which provides a theoretical basis for precise interventions in immune activation-driven diseases such as HFpEF. However, its role in signaling differentiation in different macrophage subtypes still needs to be further explored.

Corynoline (COR), an isoquinoline alkaloid derived from *Corydalis bungeana* Herba, has anti-inflammatory and anti-fibrotic potential [166,167]. It has been found that COR treatment for 2 weeks can prevent cardiac dysfunction, fibrosis, and hypertrophy in the Ang II-induced murine model of hypertensive HF (LVEF ≥ 50%, elevated CK-MB and ANP). COR has been shown to enhance the interaction between peroxisome proliferator-activated receptor alpha (PPARα) and the NF-κB subunit p65. Thereby, COR reduced macrophage (F4/80) infiltration by activating the NF-κB signaling pathway in hypertensive HF [146].

Gentiopicroside (GPS), a key active metabolite found in *Gentiana manshurica* Kitagawa, is renowned for its superior anti-inflammatory properties [168]. A recent study in a rat model of type 2 diabetes mellitus (EF ≥ 50%, LVIDd increased) showed that after 8 weeks of treatment, GPS alleviated macrophage (CD68) infiltration and fibrosis [147]. It achieved this by binding to the MH2 domain of Smad3, thereby inhibiting Smad3 phosphorylation induced by high glucose. Smad3 is a key regulator of cardiac fibroblast activation. This process is central to the development of myocardial fibrosis and HFpEF. GPS blocks Smad3 activation, which reduces high glucose-induced inflammation and reverses myocardial fibrosis. This indicates its potential as a precision therapy for metabolic heart diseases.

Triptolide is the effective metabolite of *Tripterygium wilfordii* Hook F and has attracted attention for its cardioprotective effects, particularly in MI [169,170]. Triptolide has been shown to inhibit inflammation in pressure overload model mice caused by transverse aortic constriction (TAC). Combining the LV mass index, it can be determined that this study conforms to the HFpEF model. Evidence from the study indicated that 6 weeks of triptolide treatment reduced cardiac remodeling by inhibiting NLRP3 inflammasome and activating TGF-β/Smad3 [153]. Furthermore, triptolide suppressed macrophage (F4/80) infiltration in a dose-dependent manner.

#### 5.1.4. Promote Lymphangiogenesis

Dihydrotanshinone I (DHT) is derived from *Salvia miltiorrhiza* with the function of promoting blood circulation. Mechanistically, DHT improves cardiac function by inhibiting the activation of M1 macrophages and reducing the release of pro-inflammatory cytokines. This immunomodulatory effect is particularly relevant to HFpEF, where chronic low-grade inflammation plays a central pathogenic role [171]. A study established a myocardial IR model in rats via coronary artery ligation. The model mice showed an LVEF ≥ 50% with enlarged LVIDd and LVEDV [141]. After two weeks of DHT treatment, myocardial collagen deposition, macrophages (CD68), and apoptosis were reduced. DHT upregulated lymphangiogenesis markers, including LYVE-1, VEGFR-3, and VE-cadherin. In vitro, DHT promoted migration and tube formation of human lymphatic endothelial cells via VEGFR-3 and VE-cadherin. These results suggest that DHT may improve cardiac function in HFpEF rats after IR by enhancing lymphangiogenesis. Through suppressing inflammation and promoting lymphatic drainage, DHT may simultaneously target immune dysregulation and metabolic burden. This dual mechanism offers a novel therapeutic approach for HFpEF, especially in phenotypes characterized by inflammation and microcirculatory dysfunction.

### 5.2. TCM Formulas

TCM formulas have shown unique advantages in the treatment of HFpEF. Unlike conventional single-target therapies, TCM formulas typically exert multi-targeted effects, modulating various pathogenic mechanisms such as inflammatory responses, fibrosis, endothelial dysfunction, and metabolic disorders. Many TCM formulas have been found to modulate macrophage polarization and improve myocardial microenvironment homeostasis, ultimately contributing to enhanced cardiac function. Increasing evidence suggests that TCM can complement modern pharmacological interventions by providing holistic and individualized therapeutic strategies for HFpEF patients (Table 2).

Zhen Wu Tang (ZWT) is a TCM formula derived from the *Treatise on Febrile Diseases* (Shang Han Lun) by Zhongjing Zhang. It is composed of five botanical drugs, including *Aconitum carmichaelii* Debx. (Ranunculaceae; Aconiti Lateralis Radix Praeparata), *Poria cocos* (Schw.) Wolf (Polyporaceae; Poria), *Atractylodes macrocephala* Koidz. (Asteraceae; Atractylodis Macrocephalae Rhizoma), *Zingiber officinale* Rosc. (Zingiberaceae; Zingiberis Rhizoma Recens), and *Paeonia lactiflora* Pall. (Paeoniaceae; Paeoniae Radix Alba) [178]. They work synergistically in ZWT to warm the interior, strengthen the spleen, and promote the flow of Qi and blood. Two studies were conducted to perform secondary analysis of all current clinical studies on ZWT in the treatment of HF [179,180]. The study showed that ZWT had significant efficacy in improving ejection fraction, increasing six-minute walking distance, and elevating BNP. In an ISO-induced myocardial fibrosis murine model, with preserved LVEF (≥50%) and increased LVIDd, ZWT or captopril was administered intragastrically for 30 days. It was found that ZWT significantly improved cardiac function and reduced myocardial fibrosis [172]. Histological analysis showed reduced collagen deposition and downregulation of fibrosis-related markers, including α-SMA, collagen I, and collagen III. These findings suggest ZWT suppresses fibroblast-to-myofibroblast transition, which is the key to HFpEF-related diastolic dysfunction. The study also reported that ZWT inhibited M1 macrophage (CD86 and iNOS) activation in vivo and in LPS-stimulated RAW264.7 cells in vitro, likely via suppression of the TLR4/NF-κB pathway. Notably, ZWT’s potential advantage lies in its systemic regulatory capacity. It interacts with a variety of physiological systems, including chronic inflammation and metabolic abnormalities of intestinal and renal organs [181,182]. These systemic effects are critical in HFpEF, where multi-organ crosstalk exacerbates cardiac pathology. ZWT may be suited for treating HFpEF patients with cold-damp constitution or spleen-kidney yang deficiency in TCM terms.

Shenfu Injection (SFI) is derived from the classical TCM formula Shenfu tang. It was first recorded in the Jisheng Fang by the Song dynasty physician Yonghe Yan. It is composed of *Panax ginseng* C.A. Meyer (Araliaceae; Ginseng Radix et Rhizoma) and *Aconitum carmichaelii* Debeaux (Ranunculaceae; Aconiti Lateralis Radix Prae-parata) [183]. A total of 1 mL of SFI is extracted from 0.1 g of *Panax ginseng* C.A. Meyer and 0.2 g of *Aconitum carmichaelii* Debx. Shenfu Injection has the effects of replenishing qi, invigorating blood circulation, restoring yang, rescuing from collapse, and strengthening the heart to generate pulse. It is used to treat HF, infectious shock, and other cardiovascular diseases, and its efficacy has been confirmed in multiple studies [184,185,186,187]. In a murine model of ISO-established HF (mean LVEF ≥ 50% accompanied by elevated NT-proBNP), SFI was administered at 9 mL/kg/day for 15 days [173]. Flow cytometry confirmed that SFI significantly decreased M1 macrophages (CD86) and increased M2 macrophages (CD163) compared with Tak-242-treated controls. This shift was associated with inhibition of the TLR4/NF-κB signaling pathway. ELISA and cardiac ultrasound results confirmed that SFI reduced myocardial inflammation and improved cardiac function. These results highlight the anti-inflammatory and cardioprotective potential of SFI in the heart, particularly by restoring the M1/M2 macrophage balance.

Xinyang Tablet is a hospital preparation from the First Affiliated Hospital of Guangzhou University of Traditional Chinese Medicine. It is used to treat chronic HF, consisting of *Panax ginseng* C.A. Meyer (Araliaceae; Ginseng Radix Rubra), *Epimedium brevicornum* Maxim. (Berberidaceae; Epimedii Herba), *Astragalus membranaceus* (Fisch.) Bunge (Fabaceae; Astragali Radix), *Leonurus japonicus* Houtt. (Lamiaceae; Leonuri Herba), *Isatis tinctoria* L. (Brassicaceae; Isatidis Folium), *Lepidium apetalum* Willd. (Brassicaceae; Lepidii Apetali Semen), and *Plantago asiatica* L. (Plantaginaceae; Plantaginis Herba). Known for its warming yang and promoting diuretic effects, Xinyang Tablet has shown potential in enhancing heart function. In a small sample size clinical study, Xinyang Tablet was used to evaluate the effects on brain natriuretic peptide, ultrasensitive C-reactive protein, and cardiac function in patients with acute decompensated HF [188]. After short-term treatment, patients receiving Xinyang Tablet alongside conventional Western medicine showed improved prognosis. They also had reduced cardiac function markers and inflammatory factors compared to those receiving only Western medicine. A study investigated the mechanism by which Xinyang Tablet alleviates cardiac fibrosis. A murine model of uremic cardiomyopathy (UCM) was established using 5/6 nephrectomy, presenting with reduced E/A ratio, increased LVIDd, and preserved LVEF (≥50%), thereby recapitulating the core features of HFpEF. Mice were treated with Xinyang Tablet at 0.34 g/kg for 8 weeks. Compared to the metoprolol and sham groups, Xinyang Tablet significantly improved cardiac function and reduced myocardial apoptosis and fibrosis. Mechanistically, it suppressed macrophage (F4/80)-derived osteopontin (OPN) expression and downstream fibrotic signaling, suggesting it may attenuate HFpEF-related cardiac remodeling by blocking the OPN pathway [174]. OPN is increasingly recognized as a mediator of myocardial inflammation and fibrosis in HFpEF [189]. These findings position Xinyang Tablet as a potential modulator of the “fibrosis–inflammation axis” central to disease progression. Importantly, improvements in cardiac structure and function were achieved without adverse effects on renal function, as serum creatinine and BUN levels remained unchanged—a key consideration in the setting of cardiorenal syndromes.

Fangji Fuling tang is a classical Chinese formula originating from “Synopsis of Golden Chamber” by Zhang Zhongjing. The formula consists of *Stephania tetrandra* S. Moore (Menispermaceae; Stephaniae Tetrandrae Radix), *Poria cocos* (Schw.) Wolf (Polyporaceae; Poria), *Cinnamomum verum* J. Presl (Lauraceae; Cinnamomi Ramulus), *Astragalus mongholicus* Bunge (Fabaceae; Astragali Radix), and *Glycyrrhiza uralensis* Fisch. (Fabaceae; Glycyrrhizae Radix et Rhizoma). It is known for its ability to warm yang, promote diuresis, and tonify the qi, making it a common remedy for treating HF, kidney injury, and lower limb deep vein thrombosis. A preliminary exploratory clinical study has shown that the combined use of the Fangji Fuling tang improves various cardiac ultrasound markers, including left ventricular internal diameter shortening, left ventricular posterior wall thickness, and left ventricular end-systolic internal diameter, in patients with HF [190]. In an ISO-induced myocardial fibrosis mouse model, mice exhibited elevated CK-MB levels, indicating the formation of cardiac injury [175], which is similar to the myocardial fibrosis characteristics observed in HFpEF. After two weeks of treatment, Fangji Fuling tang reduced pro-inflammatory cytokines (TNF-α, IL-1β, IL-6), increased IL-10, and promoted the polarization of macrophages toward a reparative M2 phenotype (CD206). This results in limited tissue damage, collagen deposition, and maladaptive remodeling. By regulating macrophage plasticity and inhibiting the fibrotic signals of M1 macrophages (CD86), it redirected the immune response toward repair.

QiShenYiQi Pill (QSYQ) is a proprietary TCM developed by Tasly Pharmaceutical Group. Its formulation is based on classical theories of TCM and is refined through modern scientific extraction and processing techniques. It is used for treating cardiac dysfunction and integrates four medicinal plants: *Astragalus mongholicus* Bunge (Fabaceae; Astragali Radix), *Panax notoginseng* (Burkill) F.H. Chen (Araliaceae; Notoginseng Radix et Rhizoma), *Salvia miltiorrhiza* Bunge (Lamiaceae; Salviae Miltiorrhizae Radix et Rhizoma), and *Dalbergia odorifera* T.C. Chen (Fabaceae; Dalbergiae Odoriferae Lignum). The major active metabolites of QSYQ include astragaloside IV (AS-IV, from *Astragali Radix*), notoginsenoside R1 (R1, from *Notoginseng Radix et Rhizoma*), 3,4-dihydroxy-phenyl lactic acid (DLA, from *Salviae Miltiorrhizae Radix et Rhizoma*), and *Dalbergia odorifera* oil (DO, from *Dalbergiae Odoriferae Lignum*). QSYQ has been clinically shown to improve left ventricular diastolic function, increase BNP levels, and improve exercise tolerance in patients with HFpEF [191]. The latest prospective study suggests that QSYQ has satisfactory prognostic improvement for different types of HF [192]. A study established a rat model of pressure overload–induced cardiac hypertrophy via aortic constriction [176]. The ejection fraction of the hearts of mice with this type of model is usually preserved. Elevated serum BNP levels were detected in this study, so a preliminary diagnosis of HF can be made. QSYQ was administered for 6 weeks. It significantly reduced myocardial fibrosis and apoptosis, regulated M1 (CD80)/M2 (CD163) polarization of macrophages, and inhibited RP S19 release and TGF-β1/Smad signaling. In vitro assays confirmed that its main components—astragaloside IV, notoginsenoside R1, DLA, and *Dalbergia odorifera* oil—synergistically regulated macrophage polarization and fibroblast activation. In a murine model of HFpEF induced by a high-fat diet combined with L-NAME, QSYQ significantly improved diastolic function compared to dapagliflozin [177]. Its benefits appear to involve two main mechanisms: anti-inflammatory effects and restoration of endothelial function. QSYQ reduced infiltration of inflammatory cytokines, T cells, and monocytes, suggesting suppressed endothelial activation and immune recruitment. It also enhanced NO bioavailability by activating the NO/cGMP/PKG pathway and reducing eNOS uncoupling. This pathway is essential for vascular tone, myocardial relaxation, and limiting fibrotic signaling. In another multi-omics study, the presence of QSYQ genes regulating cardiac hypertrophy, energy metabolism, and myocardial fibrosis was further confirmed. Fatty acid metabolism may be the main mechanism by which QSYQ regulates myocardial energy metabolism in HFpEF. Furthermore, it was found that QSYQ had a reduced cardioprotective effect in HFpEF mice after RNA interference-mediated knockdown of myocardial PKG [90]. Interestingly, while QSYQ does not significantly alter macrophage (F4/80) infiltration, its modulation of endothelial inflammation may indirectly influence the inflammatory milieu in HFpEF.

## 6. Conclusions

HFpEF is characterized by a multifactorial pathophysiology involving persistent inflammatory responses, myocardial fibrosis, and microvascular dysfunction, with macrophage phenotypic switching serving as a pivotal immunoregulatory process. Unlike HFrEF, where adverse outcomes are primarily driven by the HF itself, the poor prognosis of HFpEF is closely associated with comorbidities [193]. Consequently, most available HFpEF models are comorbidity-based and, in practice, closely resemble related disease models such as myocardial hypertrophy, myocardial infarction, or diabetes [9]. As a result, the majority of studies on TCM rely on these models, with relatively few conducted in HFpEF-specific settings. Findings from related conditions demonstrating the effects of TCM interventions on macrophage polarization may provide indirect yet meaningful support for the potential of macrophage-targeted therapies in HFpEF. This review focuses on the potential mechanisms and recent advances in TCM for improving HFpEF through the modulation of macrophages. TCM interventions have been shown to influence various intracellular signaling cascades, potentially re-establishing immune homeostasis and enhancing cardiac function by balancing the M1 pro-inflammatory and M2 anti-inflammatory macrophage phenotypes.

TCM agents that modulate macrophage polarization in HFpEF can be grouped into two types: metabolites and formulas (Table 3). Metabolites such as resveratrol, vanillic acid, and leonurine have a rapid onset (around 14 days) and mainly target single pathways like NF-κB, MAPK, NLRP3, and AMPK. They primarily inhibit M1 polarization and reduce inflammation, fibrosis, and oxidative stress. The therapeutic effects of TCM metabolites in modulating macrophage polarization in HFpEF are closely linked to their pharmacokinetic profiles, particularly absorption and bioavailability. Key constituents such as resveratrol and tanshinone IIA exhibit promising immunomodulatory activity but suffer from poor oral bioavailability due to low solubility and rapid metabolism. To ensure pharmacological efficacy, it is crucial to consider dosage forms and delivery systems that improve bioavailability, and future studies should integrate pharmacokinetic evaluation to support clinical translation.

In contrast, formulas like Zhen Wu Tang, Shenfu Injection, and QiShenYiQi Pill require a longer treatment duration and affect multiple pathways, including TLR4/NF-κB, TGF-β/Smads, and PI3K/Akt/mTOR. These formulas regulate both M1 and M2 macrophages and offer broader immune, metabolic, and fibrotic modulation, reflecting their systemic and multi-targeted nature. Although many TCMs have shown beneficial effects in vitro and in animal models, their impact on macrophage polarization and the precise underlying pathways remains incompletely understood. This limits the relevance of these findings for HFpEF treatment. While macrophage polarization is recognized as a key pathogenic mechanism in HFpEF, clinical research targeting this pathway is relatively scarce, and its efficacy in real-world patients remains to be verified.

Compared with established HFpEF therapies such as SGLT2 inhibitors, TCM interventions differ in mechanisms and therapeutic targets, offering complementary rather than substitutive benefits. Preclinical and clinical studies of SGLT2i in HFpEF are already extensive, and their effects on macrophages are well documented [12], with robust clinical evidence showing outcome improvement partly via metabolic and anti-inflammatory pathways [194]. TCM, in contrast, generally exerts multi-target effects, including modulation of macrophage polarization, regulation of oxidative stress, and enhancement of microvascular function. While research on TCM in HFpEF is still at an early stage, particularly regarding macrophage-mediated mechanisms, high-quality and standardized studies may reveal synergistic potential with existing therapies, highlighting a complementary role rather than replacement.

However, clinical translation faces multiple challenges, including the lack of standardized herbal formulations and dosages, inconsistent quality control, and insufficient pharmacokinetic and safety data. The method of targeting macrophages to deliver drugs is also a technical challenge [195,196]. There is still a lack of high-quality clinical evidence, especially randomized controlled trials or large observational studies. Future studies should focus on mechanism-driven clinical research, integrating immune profiling and rigorous outcome measures to systematically evaluate the therapeutic potential of TCM targeting macrophage polarization.

Moreover, inherent challenges of TCM include low bioavailability, nonspecific tissue distribution, and complex pharmacokinetics. Some active metabolites contain substructures prone to nonspecific target binding or enzyme inhibition, which may cause false positives in in vitro high-throughput screens and are difficult to extrapolate directly to in vivo effects. The safety profiles of many TCM components remain inadequately characterized. Certain herbs in traditional formulas may cause adverse reactions and pose risks of off-target effects or drug interactions [197]. These safety concerns require thorough evaluation during preclinical and clinical stages to ensure patient safety.

## 7. Prospects

Although progress has been made, significant gaps persist in HFpEF modeling and clinical validation of current therapies. To better understand macrophage heterogeneity and function in HFpEF, the development of robust HFpEF models is crucial. Cutting-edge techniques such as single-cell RNA sequencing, spatial transcriptomics, and multi-omics integration will be instrumental in this effort. Additionally, targeted delivery methods such as nanocarriers, exosomes, and biomaterial-based platforms hold promise for enhancing cardiac-specific bioactivity of therapeutic agents. Finally, well-designed clinical trials with standardized TCM formulations and rigorous outcome measures are essential to translate preclinical insights into effective, macrophage subtype-specific treatments for HFpEF.

## Figures and Tables

**Table 1:** Studies of HFpEF with active metabolites of TCM by regulating macrophages.

Name	Structure	Source	In Vivo	Action Time	In Vitro	Targets or Related Signal Pathways	Changes in Macrophages	Ref.
Dihydrotanshinone I	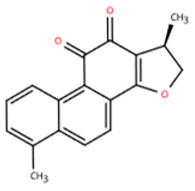	*Salvia miltiorrhiza*	Ligation of the left anterior descending coronary artery in SD rats	2 weeks	350 μM H_2_O_2_ or 6 h OGD followed by 24 h reoxygenation-induced human lymphatic endothelial cells	LYVE-1, PROX1, VEGF-C, VEGFR-3, VE-cadherin, IGF-1, IGF-1R↑	M1↓	[141]
Schisandrin B	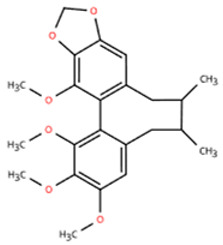	*Schisandra chinensis*	AngII (1.4 mg/kg) s.c. in C57BL/6J wild-type mice	2 weeks	1 μM AngⅡ-induced H9c2 and primary rat cardiomyocytes	(1) Myd88/TLR signaling pathway (2)*β-Mhc*, *Tgfb*, *Anp*, *α-Ska*, *Il6*, *Tnf*, *Col1a1*, *α-SkA*↓	F4/80 macrophages↓	[142]
Vanillic acid	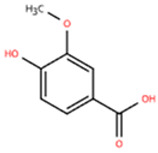	*Salvia miltiorrhiza*, Mint, *Pueraria lobata*, etc.	ISO (10 mg/kg) s.c. in C57BL/6J mice	2 weeks	-	(1) Drp1/HK1/NLRP3 signaling pathway (2) ROS, LDH, IL-1β, IL-6, IL-18, TNF-α, MDA, MPO, iNOS, Coll I, Coll III, α-SMA↓ (3) IL-4, IL-10, CAT, GSH, SOD, T-AOC, Arg-1 ↑	M1↓, M2↑	[143]
20(S)-ginsenoside Rh2	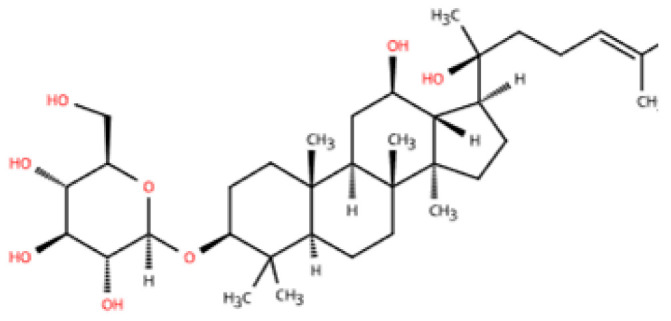	Ginseng	Ang-II infusion (1 ng/kg/min) via micro-osmotic pump implanted in C57BL/6 mice	2 weeks	Ang-II-induced NRVMs	(1) JNK/AP-1 signaling pathway (2) *Il1b*, *Il6*, *Tnfa*, TGF-β1, β-MyHC, collagen I↓	F4/80 mRNA↓	[144]
Leonurine	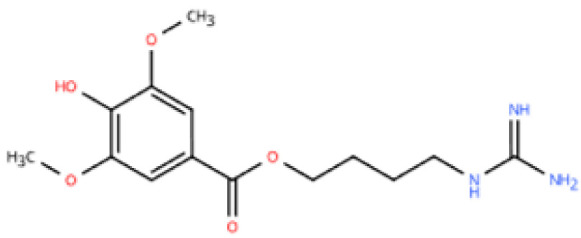	*Leonurus japonicus* Houtt	Ang-II infusion (1000 ng/kg/min) via micro-osmotic pump implanted in C57BL/6 mice	2 weeks	Ang II (1 μM)-induced H9c2 cells and NRVMs	(1) MAPK signaling pathway (2) NF-κB signaling pathway (3) *Il1b*, *Il6*, *Tnfa*, β-MyHC, collagen I, TGF-β1↓	macrophage marker F4/80↓	[145]
Corynoline	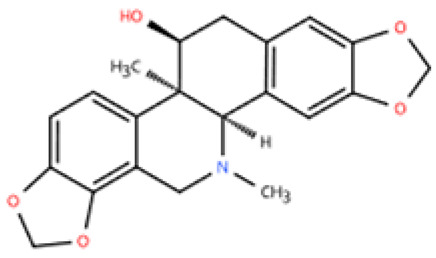	*Corydalis bungeana* Herba	Ang-II infusion (1000 ng/kg/min) via micro-osmotic pump implanted in C57BL/6 mice	2 weeks	Ang II (1 μM)-induced H9c2 cells	(1) NF-κB signaling pathway↓ (2) TGF-β1, β-MyHC, collagen I, *Il1b*, *Il6*, *Tnfa*↓ (3) PPARα↑	M1↓	[146]
Gentiopicroside	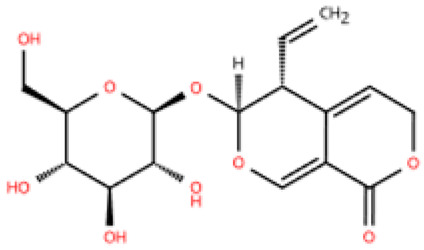	*Gentiana manshurica* Kitagawa	High-fat diet and streptozotocin (50 mg/kg) i.p. in Sprague Dawley rats	8 weeks	30 mM high glucose-induced cardiac fibroblasts	(1) *Smad3*, collagen I and III, IL-1β, IL-6, TNF-α, MDA, NOX2, NOX4↓ (2) SOD↑	M1↓	[147]
Resveratrol	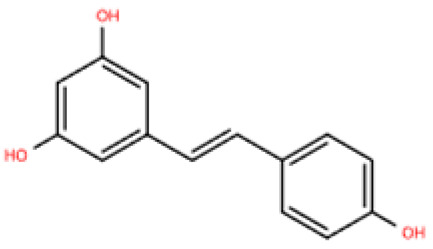	Grapes, berries, *Polygonum cuspidatum*, etc.	ISO (50 mg/kg) s.c. in BALB/c mice	1 week	50 μmol/L ISO-induced RAW264.7	(1) VEGFB/AMPK/NF-кB signaling pathway (2) Nppa, *Il6*, *Tnf*, *Ccl2*, *Ptgs2*, *Icam1*, *Vcam1*↓ (3) *Il10*, *Il1rn*↑	M1↓, M2↑	[148]
Resveratrol	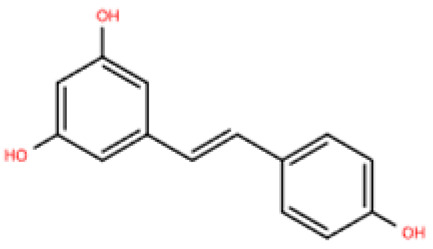	Same as above	Uninephrectomy surgery in C57BL/6 mice	4 weeks	10 ng/mL TGF-β-induced cardiac fibroblasts	(1) TGF-β/Smad3 signaling pathway (2) IL-1β, IL-6, TNF-α, *Col1a1*, *Col3a1*, *Nos2*, GSH, CAT, SOD↓ (3) Sirt1, eNOS↑	M1↓, M2↑	[149]
Cardamonin	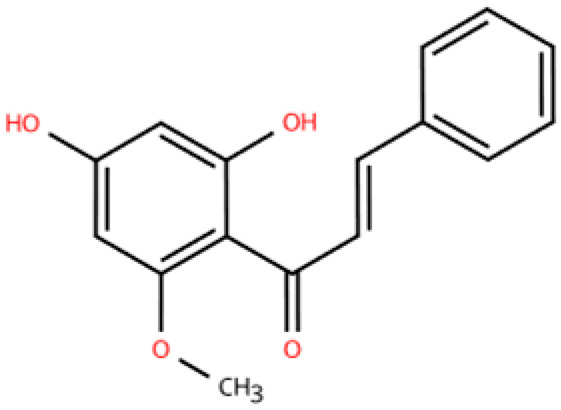	Alpinia plant	DOX (5 mg/kg) i.p. in C57BL/6J mice	4 weeks	5 μM DOX-induced HL-1	(1) Nrf2 signaling pathway↑ (2) Caspase-3, Keap1, NF-κB, MDA, ROS, TNF-α, IL-1β, IL-6, IL-18↓ (3) SOD, GSH, CAT↑	macrophage marker F4/80↓	[150]
Latifolin	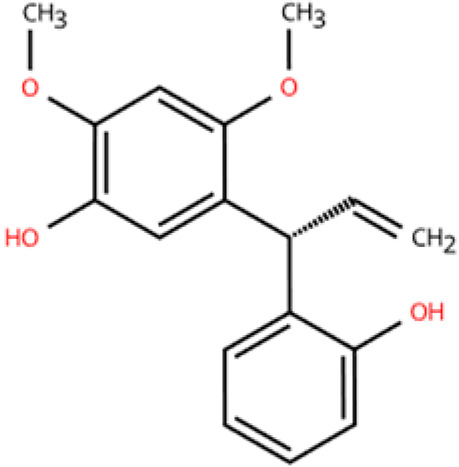	*Lignum dalbergiae odoriferae*	DOX (20 mg/kg) i.p. in C57BL/6 mice	12 days	100 ng/mL LPS and 30 ng/mL IFN-γ-induced peritoneal macrophages	(1) *Nos2*, *Il6*, *Il1b*, *Tnf*, LDH↓ (2) *Il10*, *Il4ra*↑	M1↓, M2↑	[151]
Arctigenin	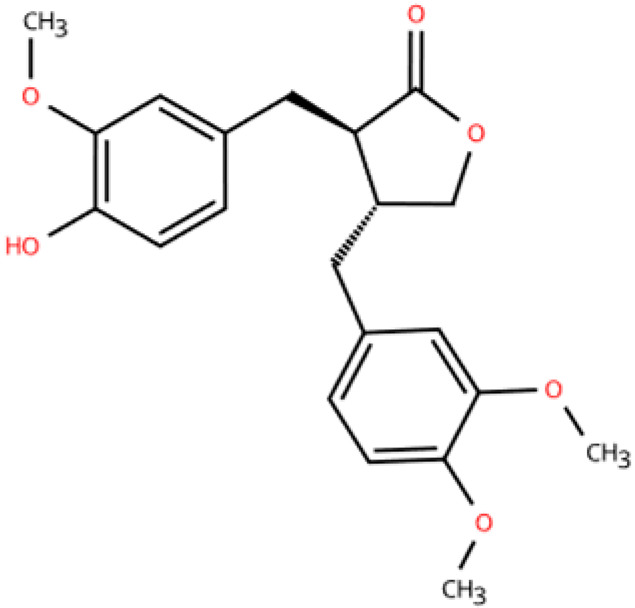	*Arctium lappa* and *Forsythia suspensa*	Coronary artery ligation in C57BL/6 mice	18 weeks	0.2 mg/mL LPS-induced RAW264.7	(1) JAK/STAT signaling pathway (2) NF-κB signaling pathway (3) NFAT5, *Il6*, *Tnfa*↓	M1,M2c↓ M2a, M2b, M2d↑	[152]
Triptolide	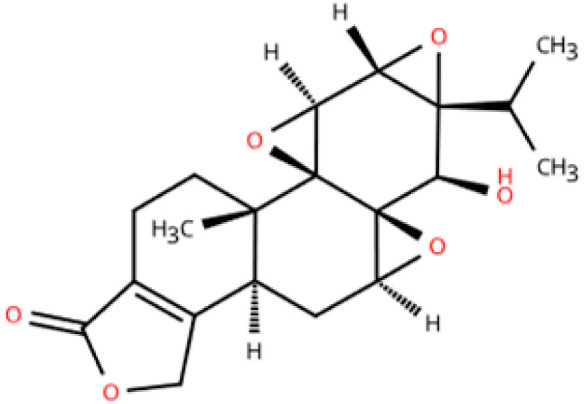	*Tripterygium wilfordii* Hook F	Transverse aortic constriction operation in C57/BL6 mice	6 weeks	-	(1) NLRP3 inflammasome↓ (2) *Il1b*, *Il18*, *Ccl2*, *Vcam1*, Collagen I and III, ASC↓ (3) TGFβ1, p-Smad3↓	macrophage marker F4/80↓	[153]

Note: ↑ indicates upregulation or increase in expression/activity following treatment. ↓ indicates downregulation or decrease in expression/activity.

**Table 2:** Studies of HFpEF with TCM formulas by regulating macrophages.

Name	Main Herbal Ingredients	In Vivo	Action Time	In Vitro	Mechanisms	Changes in Macrophages	Ref.
Zhen Wu Tang	*Aconitum carmichaelii*, *Poria cocos*, *Atractylodes macrocephala*, *Zingiber officinale*, *Paeonia lactiflora*	ISO (2.5 mg/kg) i.p. in Kunming mice	30 days	1 μg/mL LPS-induced RAW 264.7 cells	(1) TLR4/NF-κB signaling pathway (2) α-SMA, collagen I, and collagen III, iNOS↓	M1↓	[172]
Shenfu Injection	*Panax ginseng*, *Aconitum carmichaelii*	ISO (7.5 mg/kg) i.p. in C57BL/6J mice	15 days	-	(1) TLR4/NF-κB signaling pathway (2) IL-6, TNFα↓ (3) IL-10, Arg-1↑	M1↓, M2↑	[173]
Xinyang Tablet	*Panax ginseng*, *Epimedium brevicornum*, *Astragalus membranaceus*, *Leonurus japonicus*, *Isatis tinctoria*, *Lepidium apetalum*, *Plantago asiatica*	Uninephrectomy surgery in C57BL/6 mice	8 weeks	-	OPN, α-SMA, *acta2*, *col1a1*, *col2a1*, *mmp3*, *mmp9*↓	macrophage marker F4/80↓	[174]
Fangji Fuling tang	*Stephania tetrandra*, *Poria cocos*, *Cinnamomum verum*, *Astragalus mongholicus*, *Glycyrrhiza uralensis*	ISO (5 mg/kg) s.c. in C57BL/6J mice	2 weeks	-	(1) TGF-β1, TNF-α, IL-1β, IL-6, SOD, GSH↓ (2) IL-10, MDA↑	M1↓, M2↑	[175]
QiShenYiQi Pill	*Astragalus mongholicus*, *Panax notoginseng*, *Salvia miltiorrhiza*, *Dalbergia odorifera*	Ascending aortic stenosis surgery in Sprague–Dawley rats	6 weeks	1 μM Ang II-induced H9c2, 10 ng/mL TGF-β1-induced RDF, 100 ng/ml	(1) TGF-β1/Smads signaling pathway (2) RPS19, MCP-1, MDA, LDH, cleaved caspase-9, cleaved caspase-3, MMP2, MMP9, TIMP1, collagen I, collagen III↓ (3) FHL2, TIMP2, ATP↑	M1↓, M2↓	[176]
QiShenYiQi Pill	Same as above	L-NAME and high-fat diet for C57BL/6N male mice	4 weeks	-	(1) NO/cGMP/PKG signaling pathway (2) NF-κB, NLRP3, TNF-α, MCP-1, ROS, *Icam1*, *Vcam1*, *Sele*↓	No significant changes	[177]

Note: ↑ indicates upregulation or increase in expression/activity following treatment. ↓ indicates downregulation or decrease in expression/activity.

**Table 3:** Comparative mechanisms between TCM metabolites and formulas.

Category	Representative Agents	Action Time	Main Pathways Targeted	Macrophage Regulation	Mechanistic Features
Metabolites	Resveratrol, vanillic acid, gentiopicroside, leonurine, arctigenin	Mostly 14 days	NF-κB, MAPK, JNK/AP-1, NLRP3, TGFβ/Smads, AMPK, JAK/STAT, Nrf2	Mostly M1↓, some M2↑	Single pathway focused Mainly inhibit inflammation, fibrosis, and oxidative stress
Formulas	Zhen Wu Tang, Shenfu Injection, Xinyang Tablet, QiShenYiQi Pill, Fangji Fuling Tang	Over 14 days	TLR4/NF-κB, TGFβ/Smads, OPN, NO/cGMP/PKG, PI3K/Akt/mTOR	M1↓, M2↑ or bidirectional	Several pathways, systemic regulation across immune-metabolic-fibrotic axes

Note: ↑ indicates upregulation or increase in expression/activity following treatment. ↓ indicates downregulation or decrease in expression/activity.

## Data Availability

Not applicable.

## References

[1] Kalogeropoulos A.P., Butler J. (2022). Worsening Cardiovascular Disease Epidemiology in the United States. J. Am. Coll. Cardiol..

[2] Savarese G., Becher P.M., Lund L.H., Seferovic P., Rosano G.M.C., Coats A.J.S. (2023). Global Burden of Heart Failure: A Comprehensive and Updated Review of Epidemiology. Cardiovasc. Res..

[3] McDonagh T.A., Metra M., Adamo M., Gardner R.S., Baumbach A., Böhm M., Burri H., Butler J., Čelutkienė J., Chioncel O. (2024). 2023 Focused Update of the 2021 ESC Guidelines for the Diagnosis and Treatment of Acute and Chronic Heart Failure. Eur. J. Heart Fail..

[4] Campbell P., Rutten F.H., Lee M.M., Hawkins N.M., Petrie M.C. (2024). Heart Failure with Preserved Ejection Fraction: Everything the Clinician Needs to Know. Lancet.

[5] Khan M.S., Shahid I., Bennis A., Rakisheva A., Metra M., Butler J. (2024). Global Epidemiology of Heart Failure. Nat. Rev. Cardiol..

[6] Heidenreich P.A., Albert N.M., Allen L.A., Bluemke D.A., Butler J., Fonarow G.C., Ikonomidis J.S., Khavjou O., Konstam M.A., Maddox T.M. (2013). Forecasting the Impact of Heart Failure in the United States: A Policy Statement from the American Heart Association. Circ. Heart Fail..

[7] Mohammed S.F., Hussain S., Mirzoyev S.A., Edwards W.D., Maleszewski J.J., Redfield M.M. (2015). Coronary Microvascular Rarefaction and Myocardial Fibrosis in Heart Failure with Preserved Ejection Fraction. Circulation.

[8] Shah S.J., Borlaug B.A., Kitzman D.W., McCulloch A.D., Blaxall B.C., Agarwal R., Chirinos J.A., Collins S., Deo R.C., Gladwin M.T. (2020). Research Priorities for Heart Failure with Preserved Ejection Fraction: National Heart, Lung, and Blood Institute Working Group Summary. Circulation.

[9] Mishra S., Kass D.A. (2021). Cellular and Molecular Pathobiology of Heart Failure with Preserved Ejection Fraction. Nat. Rev. Cardiol..

[10] Alcaide P., Kallikourdis M., Emig R., Prabhu S.D. (2024). Myocardial Inflammation in Heart Failure with Reduced and Preserved Ejection Fraction. Circ. Res..

[11] Feijóo-Bandín S., Aragón-Herrera A., Otero-Santiago M., Anido-Varela L., Moraña-Fernández S., Tarazón E., Roselló-Lletí E., Portolés M., Gualillo O., González-Juanatey J.R. (2022). Role of Sodium-Glucose Co-Transporter 2 Inhibitors in the Regulation of Inflammatory Processes in Animal Models. Int. J. Mol. Sci..

[12] Rykova E.Y., Klimontov V.V., Shmakova E., Korbut A.I., Merkulova T.I., Kzhyshkowska J. (2025). Anti-Inflammatory Effects of SGLT2 Inhibitors: Focus on Macrophages. Int. J. Mol. Sci..

[13] Shah S.J., Kitzman D.W., Borlaug B.A., van Heerebeek L., Zile M.R., Kass D.A., Paulus W.J. (2016). Phenotype-Specific Treatment of Heart Failure with Preserved Ejection Fraction: A Multiorgan Roadmap. Circulation.

[14] Wang K., Sun X., Sun Y., Jiao B., Yao J., Hu Y., Deng Q., Dong J., Wang W., Wang Y. (2023). Transcriptional Regulation of Macrophages in Heart Failure. Front. Cardiovasc. Med..

[15] Moskalik A., Niderla-Bielińska J., Ratajska A. (2022). Multiple Roles of Cardiac Macrophages in Heart Homeostasis and Failure. Heart Fail. Rev..

[16] Qin D., Zhang Y., Liu F., Xu X., Jiang H., Su Z., Xia L. (2024). Spatiotemporal Development and the Regulatory Mechanisms of Cardiac Resident Macrophages: Contribution in Cardiac Development and Steady State. Acta Physiol..

[17] Chen R., Zhang H., Tang B., Luo Y., Yang Y., Zhong X., Chen S., Xu X., Huang S., Liu C. (2024). Macrophages in Cardiovascular Diseases: Molecular Mechanisms and Therapeutic Targets. Signal Transduct. Target. Ther..

[18] Rizzoni D., De Ciuceis C., Szczepaniak P., Paradis P., Schiffrin E.L., Guzik T.J. (2022). Immune System and Microvascular Remodeling in Humans. Hypertension.

[19] Yang P., Chen Z., Huang W., Zhang J., Zou L., Wang H. (2023). Communications between Macrophages and Cardiomyocytes. Cell Commun. Signal.

[20] Lanzer J.D., Wienecke L.M., Ramirez Flores R.O., Zylla M.M., Kley C., Hartmann N., Sicklinger F., Schultz J.-H., Frey N., Saez-Rodriguez J. (2024). Single-Cell Transcriptomics Reveal Distinctive Patterns of Fibroblast Activation in Heart Failure with Preserved Ejection Fraction. Basic. Res. Cardiol..

[21] Kuppe C., Ramirez Flores R.O., Li Z., Hayat S., Levinson R.T., Liao X., Hannani M.T., Tanevski J., Wünnemann F., Nagai J.S. (2022). Spatial Multi-Omic Map of Human Myocardial Infarction. Nature.

[22] Manning B.D., Toker A. (2017). AKT/PKB Signaling: Navigating the Network. Cell.

[23] Huo J.-Y., Hou C., Jia F., Xue C., Li X.-L., Yang L., Jiang W.-Y., Zhang X. (2025). Transcutaneous Auricular Vagus Nerve Stimulation Ameliorates Heart Failure with Preserved Ejection Fraction Through Regulating Macrophage Polarization Mediated by Alpha7nAChR. Cardiovasc. Drugs Ther..

[24] Lu X., Yao J., Li C., Cui L., Liu Y., Liu X., Wang G., Dong J., Deng Q., Hu Y. (2022). Shexiang Tongxin Dropping Pills Promote Macrophage Polarization-Induced Angiogenesis Against Coronary Microvascular Dysfunction via PI3K/Akt/mTORC1 Pathway. Front. Pharmacol..

[25] Wang T., Wang S., Jia X., Li C., Ma X., Tong H., Liu M., Li L. (2024). Baicalein Alleviates Cardiomyocyte Death in EAM Mice by Inhibiting the JAK-STAT1/4 Signalling Pathway. Phytomedicine.

[26] Chen S., Zeng J., Li R., Zhang Y., Tao Y., Hou Y., Yang L., Zhang Y., Wu J., Meng X. (2024). Traditional Chinese Medicine in Regulating Macrophage Polarization in Immune Response of Inflammatory Diseases. J. Ethnopharmacol..

[27] Jian X., Liu Y., Zhao Z., Zhao L., Wang D., Liu Q. (2019). The Role of Traditional Chinese Medicine in the Treatment of Atherosclerosis through the Regulation of Macrophage Activity. Biomed. Pharmacother..

[28] Gianopoulos I., Daskalopoulou S.S. (2024). Macrophage Profiling in Atherosclerosis: Understanding the Unstable Plaque. Basic. Res. Cardiol..

[29] Panico C., Felicetta A., Kunderfranco P., Cremonesi M., Salvarani N., Carullo P., Colombo F., Idini A., Passaretti M., Doro R. (2023). Single-Cell RNA Sequencing Reveals Metabolic Stress-Dependent Activation of Cardiac Macrophages in a Model of Dyslipidemia-Induced Diastolic Dysfunction. Circulation.

[30] Venkatesan T., Toumpourleka M., Niewiadomska M., Farhat K., Morris L., Elkholey K., Maryam B., Cordova A., Darby I.G., Whyte S. (2025). Vagal Stimulation Rescues HFpEF by Altering Cardiac Resident Macrophage Function. Circ. Res..

[31] Hulsmans M., Schloss M.J., Lee I.-H., Bapat A., Iwamoto Y., Vinegoni C., Paccalet A., Yamazoe M., Grune J., Pabel S. (2023). Recruited Macrophages Elicit Atrial Fibrillation. Science.

[32] DeBerge M., Shah S.J., Wilsbacher L., Thorp E.B. (2019). Macrophages in Heart Failure with Reduced versus Preserved Ejection Fraction. Trends Mol. Med..

[33] Schiattarella G.G., Rodolico D., Hill J.A. (2021). Metabolic Inflammation in Heart Failure with Preserved Ejection Fraction. Cardiovasc. Res..

[34] Jian Y., Zhou X., Shan W., Chen C., Ge W., Cui J., Yi W., Sun Y. (2023). Crosstalk between Macrophages and Cardiac Cells after Myocardial Infarction. Cell Commun. Signal.

[35] Chen R., Zhang S., Liu F., Xia L., Wang C., Sandoghchian Shotorbani S., Xu H., Chakrabarti S., Peng T., Su Z. (2023). Renewal of Embryonic and Neonatal-Derived Cardiac-Resident Macrophages in Response to Environmental Cues Abrogated Their Potential to Promote Cardiomyocyte Proliferation via Jagged-1–Notch1. Acta Pharm. Sin. B.

[36] Zuo W., Sun R., Ji Z., Ma G. (2023). Macrophage-Driven Cardiac Inflammation and Healing: Insights from Homeostasis and Myocardial Infarction. Cell Mol. Biol. Lett..

[37] Bajpai G., Bredemeyer A., Li W., Zaitsev K., Koenig A.L., Lokshina I., Mohan J., Ivey B., Hsiao H.-M., Weinheimer C. (2019). Tissue Resident CCR2- and CCR2+ Cardiac Macrophages Differentially Orchestrate Monocyte Recruitment and Fate Specification Following Myocardial Injury. Circ. Res..

[38] Chakarov S., Lim H.Y., Tan L., Lim S.Y., See P., Lum J., Zhang X.-M., Foo S., Nakamizo S., Duan K. (2019). Two Distinct Interstitial Macrophage Populations Coexist across Tissues in Specific Subtissular Niches. Science.

[39] Doddapattar P., Dev R., Ghatge M., Patel R.B., Jain M., Dhanesha N., Lentz S.R., Chauhan A.K. (2022). Myeloid Cell PKM2 Deletion Enhances Efferocytosis and Reduces Atherosclerosis. Circ. Res..

[40] Peet C., Ivetic A., Bromage D.I., Shah A.M. (2020). Cardiac Monocytes and Macrophages after Myocardial Infarction. Cardiovasc. Res..

[41] Ma Y., Mouton A.J., Lindsey M.L. (2018). Cardiac Macrophage Biology in the Steady-State Heart, the Aging Heart, and Following Myocardial Infarction. Transl. Res..

[42] Wang T., Wang X., Ren W., Sun Z., Zhang Y., Wu N., Diao H. (2024). Cardiomyocyte Proliferation: Advances and Insights in Macrophage-Targeted Therapy for Myocardial Injury. Genes. Dis..

[43] Niskala A., Heijman J., Dobrev D., Jespersen T., Saljic A. (2024). Targeting the NLRP3 Inflammasome Signalling for the Management of Atrial Fibrillation. Br. J. Pharmacol..

[44] Dobrev D., Heijman J., Hiram R., Li N., Nattel S. (2023). Inflammatory Signalling in Atrial Cardiomyocytes: A Novel Unifying Principle in Atrial Fibrillation Pathophysiology. Nat. Rev. Cardiol..

[45] Cao K., Zhu Y., Kuai Y., Chen B., Zhao Q., Yu W. (2024). Macrophage MKL1 Contributes to Cardiac Fibrosis in a Mouse Model of Myocardial Infarction. Life Sci..

[46] Merino-Merino A., Gonzalez-Bernal J., Fernandez-Zoppino D., Saez-Maleta R., Perez-Rivera J.-A. (2021). The Role of Galectin-3 and ST2 in Cardiology: A Short Review. Biomolecules.

[47] Shen S., Zhang M., Wang X., Liu Q., Su H., Sun B., Guo Z., Tian B., Gan H., Gong C. (2024). Single-Cell RNA Sequencing Reveals S100a9hi Macrophages Promote the Transition from Acute Inflammation to Fibrotic Remodeling after Myocardial Ischemia-reperfusion. Theranostics.

[48] Shen J.-L., Xie X.-J. (2020). Insight into the Pro-Inflammatory and Profibrotic Role of Macrophage in Heart Failure with Preserved Ejection Fraction. J. Cardiovasc. Pharmacol..

[49] Shen S.-C., Xu J., Cheng C., Xiang X.-J., Hong B.-Y., Zhang M., Gong C., Ma L.-K. (2024). Macrophages Promote the Transition from Myocardial Ischemia Reperfusion Injury to Cardiac Fibrosis in Mice through GMCSF/CCL2/CCR2 and Phenotype Switching. Acta Pharmacol. Sin..

[50] Amrute J.M., Luo X., Penna V., Yang S., Yamawaki T., Hayat S., Bredemeyer A., Jung I.-H., Kadyrov F.F., Heo G.S. (2024). Targeting Immune-Fibroblast Cell Communication in Heart Failure. Nature.

[51] Paulus W.J., Tschöpe C. (2013). A Novel Paradigm for Heart Failure with Preserved Ejection Fraction: Comorbidities Drive Myocardial Dysfunction and Remodeling through Coronary Microvascular Endothelial Inflammation. J. Am. Coll. Cardiol..

[52] Matsiukevich D., Kovacs A., Li T., Kokkonen-Simon K., Matkovich S.J., Oladipupo S.S., Ornitz D.M. (2023). Characterization of a Robust Mouse Model of Heart Failure with Preserved Ejection Fraction. Am. J. Physiol. Heart Circ. Physiol..

[53] Meagher P., Adam M., Civitarese R., Bugyei-Twum A., Connelly K.A. (2018). Heart Failure with Preserved Ejection Fraction in Diabetes: Mechanisms and Management. Can. J. Cardiol..

[54] Takeda N., Manabe I. (2011). Cellular Interplay between Cardiomyocytes and Nonmyocytes in Cardiac Remodeling. Int. J. Inflam..

[55] Tromp J., Lim S.L., Tay W.T., Teng T.-H.K., Chandramouli C., Ouwerkerk W., Wander G.S., Sawhney J.P.S., Yap J., MacDonald M.R. (2019). Microvascular Disease in Patients with Diabetes with Heart Failure and Reduced Ejection Versus Preserved Ejection Fraction. Diabetes Care.

[56] Simmonds S.J., Cuijpers I., Heymans S., Jones E.A.V. (2020). Cellular and Molecular Differences between HFpEF and HFrEF: A Step Ahead in an Improved Pathological Understanding. Cells.

[57] Westermann D., Lindner D., Kasner M., Zietsch C., Savvatis K., Escher F., von Schlippenbach J., Skurk C., Steendijk P., Riad A. (2011). Cardiac Inflammation Contributes to Changes in the Extracellular Matrix in Patients with Heart Failure and Normal Ejection Fraction. Circ. Heart Fail.

[58] Hulsmans M., Sager H.B., Roh J.D., Valero-Muñoz M., Houstis N.E., Iwamoto Y., Sun Y., Wilson R.M., Wojtkiewicz G., Tricot B. (2018). Cardiac Macrophages Promote Diastolic Dysfunction. J. Exp. Med..

[59] Glezeva N., Voon V., Watson C., Horgan S., McDonald K., Ledwidge M., Baugh J. (2015). Exaggerated Inflammation and Monocytosis Associate with Diastolic Dysfunction in Heart Failure with Preserved Ejection Fraction: Evidence of M2 Macrophage Activation in Disease Pathogenesis. J. Card. Fail..

[60] O’Rourke S.A., Dunne A., Monaghan M.G. (2019). The Role of Macrophages in the Infarcted Myocardium: Orchestrators of ECM Remodeling. Front. Cardiovasc. Med..

[61] Yuan X., Braun T. (2017). Multimodal Regulation of Cardiac Myocyte Proliferation. Circ. Res..

[62] Mongirdienė A., Liobikas J. (2022). Phenotypic and Functional Heterogeneity of Monocyte Subsets in Chronic Heart Failure Patients. Biology.

[63] Chen P., Pan Y., Ning X., Shi X., Zhong J., Fan X., Li W., Teng Y., Liu X., Yu B. (2023). Targeted Heart Repair by Tβ4-Loaded Cardiac-Resident Macrophage-Derived Extracellular Vesicles Modified with Monocyte Membranes. Acta Biomater..

[64] Zhang R., Wang M., Lang Y., Zhang J., Wang Y., Zheng H., Zheng Y., Zhou B. (2025). Subepicardial Adipose Genes Contribute to the Deterioration of Heart Failure Preserved Ejection Fraction. Front. Cardiovasc. Med..

[65] Grune J., Lewis A.J.M., Yamazoe M., Hulsmans M., Rohde D., Xiao L., Zhang S., Ott C., Calcagno D.M., Zhou Y. (2022). Neutrophils Incite and Macrophages Avert Electrical Storm after Myocardial Infarction. Nat. Cardiovasc. Res..

[66] Jia D., Chen S., Bai P., Luo C., Liu J., Sun A., Ge J. (2022). Cardiac Resident Macrophage-Derived Legumain Improves Cardiac Repair by Promoting Clearance and Degradation of Apoptotic Cardiomyocytes After Myocardial Infarction. Circulation.

[67] Tan H., Li W., Pang Z., Weng X., Gao J., Chen J., Wang Q., Li Q., Yang H., Dong Z. (2024). Genetically Engineered Macrophages Co-Loaded with CD47 Inhibitors Synergistically Reconstruct Efferocytosis and Improve Cardiac Remodeling Post Myocardial Ischemia Reperfusion Injury. Adv. Health Mater..

[68] Deng Y., Xie M., Li Q., Xu X., Ou W., Zhang Y., Xiao H., Yu H., Zheng Y., Liang Y. (2021). Targeting Mitochondria-Inflammation Circuit by β-Hydroxybutyrate Mitigates HFpEF. Circ. Res..

[69] Chen J., Fu C.-Y., Shen G., Wang J., Xu L., Li H., Cao X., Zheng M.-Z., Shen Y.-L., Zhong J. (2022). Macrophages Induce Cardiomyocyte Ferroptosis via Mitochondrial Transfer. Free Radic. Biol. Med..

[70] Nicolás-Ávila J.A., Pena-Couso L., Muñoz-Cánoves P., Hidalgo A. (2022). Macrophages, Metabolism and Heterophagy in the Heart. Circ. Res..

[71] Nicolás-Ávila J.A., Lechuga-Vieco A.V., Esteban-Martínez L., Sánchez-Díaz M., Díaz-García E., Santiago D.J., Rubio-Ponce A., Li J.L., Balachander A., Quintana J.A. (2020). A Network of Macrophages Supports Mitochondrial Homeostasis in the Heart. Cell.

[72] Gao J., Huang C., Kong L., Zhou W., Sun M., Wei T., Shen W. (2023). SIRT3 Regulates Clearance of Apoptotic Cardiomyocytes by Deacetylating Frataxin. Circ. Res..

[73] Cai S., Zhao M., Zhou B., Yoshii A., Bugg D., Villet O., Sahu A., Olson G.S., Davis J., Tian R. (2023). Mitochondrial Dysfunction in Macrophages Promotes Inflammation and Suppresses Repair after Myocardial Infarction. J. Clin. Investig..

[74] Sugita J., Fujiu K., Nakayama Y., Matsubara T., Matsuda J., Oshima T., Liu Y., Maru Y., Hasumi E., Kojima T. (2021). Cardiac Macrophages Prevent Sudden Death during Heart Stress. Nat. Commun..

[75] Hulsmans M., Clauss S., Xiao L., Aguirre A.D., King K.R., Hanley A., Hucker W.J., Wülfers E.M., Seemann G., Courties G. (2017). Macrophages Facilitate Electrical Conduction in the Heart. Cell.

[76] Simon-Chica A., Fernández M.C., Wülfers E.M., Lother A., Hilgendorf I., Seemann G., Ravens U., Kohl P., Schneider-Warme F. (2022). Novel Insights into the Electrophysiology of Murine Cardiac Macrophages: Relevance of Voltage-Gated Potassium Channels. Cardiovasc. Res..

[77] Kotecha D., Lam C.S.P., Van Veldhuisen D.J., Van Gelder I.C., Voors A.A., Rienstra M. (2016). Heart Failure with Preserved Ejection Fraction and Atrial Fibrillation: Vicious Twins. J. Am. Coll. Cardiol..

[78] Oraii A., McIntyre W.F., Parkash R., Kowalik K., Razeghi G., Benz A.P., Belley-Côté E.P., Conen D., Connolly S.J., Tang A.S.L. (2024). Atrial Fibrillation Ablation in Heart Failure with Reduced vs Preserved Ejection Fraction: A Systematic Review and Meta-Analysis. JAMA Cardiol..

[79] Ninni S., Dombrowicz D., de Winther M., Staels B., Montaigne D., Nattel S. (2024). Genetic Factors Altering Immune Responses in Atrial Fibrillation: JACC Review Topic of the Week. J. Am. Coll. Cardiol..

[80] Yang Y., Fan A., Lin H., Wang X., Yang K., Zhang H., Fan G., Li L. (2025). Role of Macrophages in Cardiac Arrhythmias: Pathogenesis and Therapeutic Perspectives. Int. Immunopharmacol..

[81] Lin H., Liu H., Xi H., Li D., Jiang P., Wang Y., Cheng S., Jiang H., Deng H., Zhou X. (2025). Oxygen-Independent Photodynamic Therapy-Mediated Selective Consumption of M1 Macrophage Against Ventricular Arrhythmias via Sympathetic Neuromodulation. Small.

[82] Wilck N., Markó L., Balogh A., Kräker K., Herse F., Bartolomaeus H., Szijártó I.A., Gollasch M., Reichhart N., Strauss O. (2018). Nitric Oxide-Sensitive Guanylyl Cyclase Stimulation Improves Experimental Heart Failure with Preserved Ejection Fraction. JCI Insight.

[83] Mollace R., Scarano F., Bava I., Carresi C., Maiuolo J., Tavernese A., Gliozzi M., Musolino V., Muscoli S., Palma E. (2023). Modulation of the Nitric Oxide/cGMP Pathway in Cardiac Contraction and Relaxation: Potential Role in Heart Failure Treatment. Pharmacol. Res..

[84] McDonagh T.A., Metra M., Adamo M., Gardner R.S., Baumbach A., Böhm M., Burri H., Butler J., Čelutkienė J., Chioncel O. (2021). 2021 ESC Guidelines for the Diagnosis and Treatment of Acute and Chronic Heart Failure. Eur. Heart J..

[85] Redfield M.M., Borlaug B.A. (2023). Heart Failure with Preserved Ejection Fraction: A Review. JAMA.

[86] Xu L., Nagata N., Nagashimada M., Zhuge F., Ni Y., Chen G., Mayoux E., Kaneko S., Ota T. (2017). SGLT2 Inhibition by Empagliflozin Promotes Fat Utilization and Browning and Attenuates Inflammation and Insulin Resistance by Polarizing M2 Macrophages in Diet-Induced Obese Mice. EBioMedicine.

[87] Zhang N., Feng B., Ma X., Sun K., Xu G., Zhou Y. (2019). Dapagliflozin Improves Left Ventricular Remodeling and Aorta Sympathetic Tone in a Pig Model of Heart Failure with Preserved Ejection Fraction. Cardiovasc. Diabetol..

[88] Tang J., Ding Y., Chen W., Shi J., Zhang C., Zhao X., Li J., Han Z., Chen X. (2025). VASP Knockdown Ameliorates Lipopolysaccharide-Induced Acute Lung Injury with Inhibition of M1 Macrophage Polarization Through the cGMP-PKG Signaling Pathway. Inflammation.

[89] Chen L., Zhou X., Deng Y., Yang Y., Chen X., Chen Q., Liu Y., Fu X., Kwan H.Y., You Y. (2023). Zhenwu Decoction Ameliorates Cardiac Hypertrophy through Activating sGC (Soluble Guanylate Cyclase)—cGMP (Cyclic Guanosine Monophosphate)—PKG (Protein Kinase G) Pathway. J. Ethnopharmacol..

[90] Huang Y., Zhang K., Wang X., Guo K., Li X., Chen F., Du R., Li S., Li L., Yang Z. (2023). Multi-Omics Approach for Identification of Molecular Alterations of QiShenYiQi Dripping Pills in Heart Failure with Preserved Ejection Fraction. J. Ethnopharmacol..

[91] Shan X., Ji Z., Wang B., Zhang Y., Dong H., Jing W., Zhou Y., Hu P., Cui Y., Li Z. (2024). Riboflavin Kinase Binds and Activates Inducible Nitric Oxide Synthase to Reprogram Macrophage Polarization. Redox Biol..

[92] Wu K.K.-L., Xu X., Wu M., Li X., Hoque M., Li G.H.Y., Lian Q., Long K., Zhou T., Piao H. (2024). MDM2 Induces Pro-Inflammatory and Glycolytic Responses in M1 Macrophages by Integrating iNOS-Nitric Oxide and HIF-1α Pathways in Mice. Nat. Commun..

[93] Guo Y., Wen J., He A., Qu C., Peng Y., Luo S., Wang X. (2023). iNOS Contributes to Heart Failure with Preserved Ejection Fraction through Mitochondrial Dysfunction and Akt S-Nitrosylation. J. Adv. Res..

[94] Mallat Z., Gojova A., Marchiol-Fournigault C., Esposito B., Kamaté C., Merval R., Fradelizi D., Tedgui A. (2001). Inhibition of Transforming Growth Factor-β Signaling Accelerates Atherosclerosis and Induces an Unstable Plaque Phenotype in Mice. Circ. Res..

[95] Yang S., Yuan H.-Q., Hao Y.-M., Ren Z., Qu S.-L., Liu L.-S., Wei D.-H., Tang Z.-H., Zhang J.-F., Jiang Z.-S. (2020). Macrophage Polarization in Atherosclerosis. Clin. Chim. Acta.

[96] Bielecka-Dabrowa A., Sakowicz A., Misztal M., von Haehling S., Ahmed A., Pietrucha T., Rysz J., Banach M. (2016). Differences in Biochemical and Genetic Biomarkers in Patients with Heart Failure of Various Etiologies. Int. J. Cardiol..

[97] Boichenko V., Noakes V.M., Reilly-O’Donnell B., Luciani G.B., Emanueli C., Martelli F., Gorelik J. (2025). Circulating Non-Coding RNAs as Indicators of Fibrosis and Heart Failure Severity. Cells.

[98] Tuleta I., Hanna A., Humeres C., Aguilan J.T., Sidoli S., Zhu F., Frangogiannis N.G. (2024). Fibroblast-Specific TGF-β Signaling Mediates Cardiac Dysfunction, Fibrosis, and Hypertrophy in Obese Diabetic Mice. Cardiovasc. Res..

[99] Chen G., Xu H., Xu T., Ding W., Zhang G., Hua Y., Wu Y., Han X., Xie L., Liu B. (2022). Calycosin Reduces Myocardial Fibrosis and Improves Cardiac Function in Post-Myocardial Infarction Mice by Suppressing TGFBR1 Signaling Pathways. Phytomedicine.

[100] Yao Y., Hu C., Song Q., Li Y., Da X., Yu Y., Li H., Clark I.M., Chen Q., Wang Q.K. (2019). ADAMTS16 Activates Latent TGF-β, Accentuating Fibrosis and Dysfunction of the Pressure-Overloaded Heart. Cardiovasc. Res..

[101] Chowkwale M., Lindsey M.L., Saucerman J.J. (2023). Intercellular Model Predicts Mechanisms of Inflammation-Fibrosis Coupling after Myocardial Infarction. J. Physiol..

[102] Wang L., Li Y., Wang X., Wang P., Essandoh K., Cui S., Huang W., Mu X., Liu Z., Wang Y. (2020). GDF3 Protects Mice against Sepsis-Induced Cardiac Dysfunction and Mortality by Suppression of Macrophage Pro-Inflammatory Phenotype. Cells.

[103] Frangogiannis N.G. (2022). Transforming Growth Factor-β in Myocardial Disease. Nat. Rev. Cardiol..

[104] Khalil H., Kanisicak O., Prasad V., Correll R.N., Fu X., Schips T., Vagnozzi R.J., Liu R., Huynh T., Lee S.-J. (2017). Fibroblast-Specific TGF-β-Smad2/3 Signaling Underlies Cardiac Fibrosis. J. Clin. Investig..

[105] Shi Y., Liu C., Xiong S., Yang L., Yang C., Qiao W., Liu Y., Liu S., Liu J., Dong G. (2023). Ling-Gui-Qi-Hua Formula Alleviates Left Ventricular Myocardial Fibrosis in Rats with Heart Failure with Preserved Ejection Fraction by Blocking the Transforming Growth Factor-Β1 /Smads Signaling Pathway. J. Ethnopharmacol..

[106] Murphy S.P., Kakkar R., McCarthy C.P., Januzzi J.L. (2020). Inflammation in Heart Failure: JACC State-of-the-Art Review. J. Am. Coll. Cardiol..

[107] Cao X. (2016). Self-Regulation and Cross-Regulation of Pattern-Recognition Receptor Signalling in Health and Disease. Nat. Rev. Immunol..

[108] Dai Y., Wang S., Chang S., Ren D., Shali S., Li C., Yang H., Huang Z., Ge J. (2020). M2 Macrophage-Derived Exosomes Carry microRNA-148a to Alleviate Myocardial Ischemia/Reperfusion Injury via Inhibiting TXNIP and the TLR4/NF-κB/NLRP3 Inflammasome Signaling Pathway. J. Mol. Cell. Cardiol..

[109] Yang Y., Lv J., Jiang S., Ma Z., Wang D., Hu W., Deng C., Fan C., Di S., Sun Y. (2016). The Emerging Role of Toll-like Receptor 4 in Myocardial Inflammation. Cell Death Dis..

[110] Yang D., Yang L., Cai J., Hu X., Li H., Zhang X., Zhang X., Chen X., Dong H., Nie H. (2021). A Sweet Spot for Macrophages: Focusing on Polarization. Pharmacol. Res..

[111] Deng T., Hu B., Wang X., Ding S., Lin L., Yan Y., Peng X., Zheng X., Liao M., Jin Y. (2022). TRAF6 Autophagic Degradation by Avibirnavirus VP3 Inhibits Antiviral Innate Immunity via Blocking NFKB/NF-κB Activation. Autophagy.

[112] Liu T., Zhang L., Joo D., Sun S.-C. (2017). NF-κB Signaling in Inflammation. Signal Transduct. Target. Ther..

[113] Guo Q., Jin Y., Chen X., Ye X., Shen X., Lin M., Zeng C., Zhou T., Zhang J. (2024). NF-κB in Biology and Targeted Therapy: New Insights and Translational Implications. Signal Transduct. Target. Ther..

[114] Zhou J., Wang B., Wang M., Zha Y., Lu S., Zhang F., Peng Y., Duan Y., Zhong D., Zhang S. (2024). Daucosterol Alleviates Heart Failure with Preserved Ejection Fraction through Activating PPARα Pathway. Heliyon.

[115] Hao J.-M., Sun P., Zeng Y., Zhang H., Zhang Z.-D., Chang L.-P., Hou Y.-L. (2023). Qiliqiangxin Capsule Improves Cardiac Remodeling in Rats with DOCA-Salt-Induced Diastolic Dysfunction. Eur. Rev. Med. Pharmacol. Sci..

[116] Guo H., Callaway J.B., Ting J.P.-Y. (2015). Inflammasomes: Mechanism of Action, Role in Disease, and Therapeutics. Nat. Med..

[117] Mangan M.S.J., Olhava E.J., Roush W.R., Seidel H.M., Glick G.D., Latz E. (2018). Targeting the NLRP3 Inflammasome in Inflammatory Diseases. Nat. Rev. Drug Discov..

[118] Latz E., Xiao T.S., Stutz A. (2013). Activation and Regulation of the Inflammasomes. Nat. Rev. Immunol..

[119] Toldo S., Mezzaroma E., Buckley L.F., Potere N., Di Nisio M., Biondi-Zoccai G., Van Tassell B.W., Abbate A. (2022). Targeting the NLRP3 Inflammasome in Cardiovascular Diseases. Pharmacol. Ther..

[120] Zhao P., Zhou W., Zhang Y., Li J., Zhao Y., Pan L., Shen Z., Chen W., Hui J. (2020). Aminooxyacetic Acid Attenuates Post-Infarct Cardiac Dysfunction by Balancing Macrophage Polarization through Modulating Macrophage Metabolism in Mice. J. Cell Mol. Med..

[121] Golino M., Moroni F., Carbone S., Corna G., Trankle C., Billingsley H.E., Del Buono M.G., Talasaz A.H., Thomas G.K., De Ponti R. (2023). Differential Response to Interleukin-1 Blockade with Anakinra on Cardiorespiratory Fitness in Patients with Heart Failure with Preserved Ejection Fraction Stratified According to Left Ventricular Ejection Fraction. J. Am. Heart Assoc..

[122] Van Tassell B.W., Arena R., Biondi-Zoccai G., Canada J.M., Oddi C., Abouzaki N.A., Jahangiri A., Falcao R.A., Kontos M.C., Shah K.B. (2014). Effects of Interleukin-1 Blockade with Anakinra on Aerobic Exercise Capacity in Patients with Heart Failure and Preserved Ejection Fraction (from the D-HART Pilot Study). Am. J. Cardiol..

[123] Van Tassell B.W., Trankle C.R., Canada J.M., Carbone S., Buckley L., Kadariya D., Del Buono M.G., Billingsley H., Wohlford G., Viscusi M. (2018). IL-1 Blockade in Patients with Heart Failure with Preserved Ejection Fraction. Circ. Heart Fail..

[124] Zhang F.-S., He Q.-Z., Qin C.H., Little P.J., Weng J.-P., Xu S.-W. (2022). Therapeutic Potential of Colchicine in Cardiovascular Medicine: A Pharmacological Review. Acta Pharmacol. Sin..

[125] Shen S., Duan J., Hu J., Qi Y., Kang L., Wang K., Chen J., Wu X., Xu B., Gu R. (2022). Colchicine Alleviates Inflammation and Improves Diastolic Dysfunction in Heart Failure Rats with Preserved Ejection Fraction. Eur. J. Pharmacol..

[126] Linton M.F., Moslehi J.J., Babaev V.R. (2019). Akt Signaling in Macrophage Polarization, Survival, and Atherosclerosis. Int. J. Mol. Sci..

[127] Covarrubias A.J., Aksoylar H.I., Horng T. (2015). Control of Macrophage Metabolism and Activation by mTOR and Akt Signaling. Semin. Immunol..

[128] Ieronymaki E., Daskalaki M.G., Lyroni K., Tsatsanis C. (2019). Insulin Signaling and Insulin Resistance Facilitate Trained Immunity in Macrophages Through Metabolic and Epigenetic Changes. Front. Immunol..

[129] Morris G., Gevezova M., Sarafian V., Maes M. (2022). Redox Regulation of the Immune Response. Cell Mol. Immunol..

[130] You Z., Yang Z., Cao S., Deng S., Chen Y. (2022). The Novel KLF4/BIG1 Regulates LPS-Mediated Neuro-Inflammation and Migration in BV2 Cells via PI3K/Akt/NF-kB Signaling Pathway. Neuroscience.

[131] Fruman D.A., Chiu H., Hopkins B.D., Bagrodia S., Cantley L.C., Abraham R.T. (2017). The PI3K Pathway in Human Disease. Cell.

[132] McClain K.L., Bigenwald C., Collin M., Haroche J., Marsh R.A., Merad M., Picarsic J., Ribeiro K.B., Allen C.E. (2021). Histiocytic Disorders. Nat. Rev. Dis. Primers.

[133] Shen S., Huang Z., Lin L., Fang Z., Li W., Luo W., Wu G., Huang Z., Liang G. (2023). Tussilagone Attenuates Atherosclerosis through Inhibiting MAPKs-Mediated Inflammation in Macrophages. Int. Immunopharmacol..

[134] Wang J., Wang J., Zhong J., Liu H., Li W., Chen M., Xu L., Zhang W., Zhang Z., Wei Z. (2024). LRG1 Promotes Atherosclerosis by Inducing Macrophage M1-like Polarization. Proc. Natl. Acad. Sci. USA.

[135] Liu C., Long Q., Yang H., Yang H., Tang Y., Liu B., Zhou Z., Yuan J. (2024). Sacubitril/Valsartan Inhibits M1 Type Macrophages Polarization in Acute Myocarditis by Targeting C-Type Natriuretic Peptide. Biomed. Pharmacother..

[136] Yuan L., Bu S., Du M., Wang Y., Ju C., Huang D., Xu W., Tan X., Liang M., Deng S. (2023). RNF207 Exacerbates Pathological Cardiac Hypertrophy via Post-Translational Modification of TAB1. Cardiovasc. Res..

[137] Hu B., Xu L., Li Y., Bai X., Xing M., Cao Q., Liang H., Song S., Ji A. (2020). A peptide inhibitor of macrophage migration in atherosclerosis purified from the leech *Whitmania pigra*. J. Ethnopharmacol..

[138] Qin Y.-Y., Huang X.-R., Zhang J., Wu W., Chen J., Wan S., Yu X.-Y., Lan H.-Y. (2022). Neuropeptide Y Attenuates Cardiac Remodeling and Deterioration of Function Following Myocardial Infarction. Mol. Ther..

[139] Tian J., Li W., Zeng L., Li Y., Du J., Li Y., Li B., Su G. (2024). HBI-8000 Improves Heart Failure with Preserved Ejection Fraction via the TGF-Β1/MAPK Signalling Pathway. J. Cell Mol. Med..

[140] Li S., Shi Y., Yuan S., Ruan J., Pan H., Ma M., Huang G., Ji Q., Zhong Y., Jiang T. (2024). Inhibiting the MAPK Pathway Improves Heart Failure with Preserved Ejection Fraction Induced by Salt-Sensitive Hypertension. Biomed. Pharmacother..

[141] Wang Y., Zhu Y., Meng W., Zheng Y., Guan X., Shao C., Li X., Hu D., Wang M., Guo H. (2025). Dihydrotanshinone I Improves Cardiac Function by Promoting Lymphangiogenesis after Myocardial Ischemia-Reperfusion Injury. Eur. J. Pharmacol..

[142] Xu S., Hu C., Han J., Luo W., Huang L., Jiang Y., Samorodov A.V., Wang Y., Huang J. (2024). Schisandrin B Alleviates Angiotensin II-Induced Cardiac Inflammatory Remodeling by Inhibiting the Recruitment of MyD88 to TLRs in Mouse Cardiomyocytes. Int. Immunopharmacol..

[143] He H., Wu M., Xu J., Xu Q., Wan F., Zhong H., Zhang J., Zhou G., Qin H., Li H. (2025). Mechanism of Vanillic Acid against Cardiac Fibrosis Induced by Isoproterenol in Mice Based on Drp1/HK1/NLRP3 and Mitochondrial Apoptosis Signaling Pathways. Zhongguo Zhong yao za zhi= Zhongguo zhongyao zazhi= China J. Chin. Mater. Medica.

[144] Yu T., Xu J., Wang Q., Han X., Tu Y., Wang Y., Luo W., Wang M., Liang G. (2023). 20(S)-Ginsenoside Rh2 Inhibits Angiotensin-2 Mediated Cardiac Remodeling and Inflammation Associated with Suppression of the JNK/AP-1 Pathway. Biomed. Pharmacother..

[145] Shen S., Wu G., Luo W., Li W., Li X., Dai C., Huang W., Liang G. (2023). Leonurine Attenuates Angiotensin II-Induced Cardiac Injury and Dysfunction via Inhibiting MAPK and NF-κB Pathway. Phytomedicine.

[146] Wang M., Luo W., Yu T., Liang S., Sun J., Zhang Y., Han X., Long X., Liang G., Li G. (2022). Corynoline Protects Ang II-Induced Hypertensive Heart Failure by Increasing PPARα and Inhibiting NF-κB Pathway. Biomed. Pharmacother..

[147] Zou X., Zhang Y., Pan Z., Hu X., Xu Y., Huang Z., Sun Z., Yuan M., Shi J., Huang P. (2022). Gentiopicroside Alleviates Cardiac Inflammation and Fibrosis in T2DM Rats through Targeting Smad3 Phosphorylation. Phytomedicine.

[148] Li Y., Feng L., Li G., An J., Zhang S., Li J., Liu J., Ren J., Yang L., Qi Z. (2020). Resveratrol Prevents ISO-Induced Myocardial Remodeling Associated with Regulating Polarization of Macrophages through VEGF-B/AMPK/NF-kB Pathway. Int. Immunopharmacol..

[149] Zhang L., Chen J., Yan L., He Q., Xie H., Chen M. (2021). Resveratrol Ameliorates Cardiac Remodeling in a Murine Model of Heart Failure with Preserved Ejection Fraction. Front. Pharmacol..

[150] Qi W., Boliang W., Xiaoxi T., Guoqiang F., Jianbo X., Gang W. (2020). Cardamonin Protects against Doxorubicin-Induced Cardiotoxicity in Mice by Restraining Oxidative Stress and Inflammation Associated with Nrf2 signalingCardamonin. Biomed. Pharmacother..

[151] Zhang N., Shou B., Chen L., Lai X., Luo Y., Meng X., Liu R. (2020). Cardioprotective Effects of Latifolin Against Doxorubicin-Induced Cardiotoxicity by Macrophage Polarization in Mice. J. Cardiovasc. Pharmacol..

[152] Ni S.-H., Sun S.-N., Zhou Z., Li Y., Huang Y.-S., Li H., Wang J.-J., Xiao W., Xian S.-X., Yang Z.-Q. (2020). Arctigenin Alleviates Myocardial Infarction Injury through Inhibition of the NFAT5-Related Inflammatory Phenotype of Cardiac Macrophages/Monocytes in Mice. Lab. Investig..

[153] Li R., Lu K., Wang Y., Chen M., Zhang F., Shen H., Yao D., Gong K., Zhang Z. (2017). Triptolide Attenuates Pressure Overload-Induced Myocardial Remodeling in Mice via the Inhibition of NLRP3 Inflammasome Expression. Biochem. Biophys. Res. Commun..

[154] Zhu W., Luo W., Han J., Zhang Q., Ji L., Samorodov A.V., Pavlov V.N., Zhuang Z., Yang D., Yin L. (2023). Schisandrin B Protects against LPS-Induced Inflammatory Lung Injury by Targeting MyD88. Phytomedicine.

[155] Lin S., Cui J., Li X., Chen S., Gao K., Mei X. (2024). Modified ZIF-8 Nanoparticles for Targeted Metabolic Treatment of Acute Spinal Cord Injury. ACS Appl. Mater. Interfaces.

[156] Zhang R., Luo S., Zhao T., Wu M., Huang L., Zhang L., Huang Y., Gao H., Sun X., Gong T. (2023). Scavenger Receptor A-Mediated Nanoparticles Target M1 Macrophages for Acute Liver Injury. Asian J. Pharm. Sci..

[157] Yu X., Zhang Y., Wang J., Wang X., Chen X., Yin K., Zhu X. (2024). Leonurine Improves Atherosclerosis by Activating Foam Cell Autophagy and Metabolic Remodeling via METTL3-Mediated AKT1S1 mRNA Stability Modulation. Phytomedicine.

[158] Hyam S.R., Lee I.-A., Gu W., Kim K.-A., Jeong J.-J., Jang S.-E., Han M.J., Kim D.-H. (2013). Arctigenin Ameliorates Inflammation in Vitro and in Vivo by Inhibiting the PI3K/AKT Pathway and Polarizing M1 Macrophages to M2-like Macrophages. Eur. J. Pharmacol..

[159] Lin R., Duan J., Mu F., Bian H., Zhao M., Zhou M., Li Y., Wen A., Yang Y., Xi M. (2018). Cardioprotective Effects and Underlying Mechanism of Radix Salvia Miltiorrhiza and Lignum Dalbergia Odorifera in a Pig Chronic Myocardial Ischemia Model. Int. J. Mol. Med..

[160] Mu F., Duan J., Bian H., Yin Y., Zhu Y., Wei G., Guan Y., Wang Y., Guo C., Wen A. (2017). Cardioprotective Effects and Mechanism of Radix Salviae Miltiorrhizae and Lignum Dalbergiae Odoriferae on Rat Myocardial Ischemia/Reperfusion Injury. Mol. Med. Rep..

[161] Qin L., Tan J., Lv X., Zhang J. (2023). Vanillic Acid Alleviates Liver Fibrosis through Inhibiting Autophagy in Hepatic Stellate Cells via the MIF/CD74 Signaling Pathway. Biomed. Pharmacother..

[162] Yu D., Tang Z., Li B., Yu J., Li W., Liu Z., Tian C. (2021). Resveratrol against Cardiac Fibrosis: Research Progress in Experimental Animal Models. Molecules.

[163] Zheng X., Sun K., Liu Y., Yin X., Zhu H., Yu F., Zhao W. (2023). Resveratrol-Loaded Macrophage Exosomes Alleviate Multiple Sclerosis through Targeting Microglia. J. Control Release.

[164] Huang M., Chen Y., Lyu W., He X., Ye Z., Huang C.-Y., He X.-L., Chen X., Chen X., Zhang B. (2023). Ginsenoside Rh2 Augmented Anti-PD-L1 Immunotherapy by Reinvigorating CD8+ T Cells via Increasing Intratumoral CXCL10. Pharmacol. Res..

[165] Zhou Z., Wu S., Li Y., Shao P., Jiang J. (2025). Inhibition of Macrophage Polarization and Pyroptosis in Collagen-Induced Arthritis through MSC-Exo and Ginsenoside Rh2. Arthritis Res. Ther..

[166] Liu Y., Song M., Zhu G., Xi X., Li K., Wu C., Huang L. (2017). Corynoline Attenuates LPS-Induced Acute Lung Injury in Mice by Activating Nrf2. Int. Immunopharmacol..

[167] Wang P., Huang B., Liu Y., Tan X., Liu L., Zhang B., Li Z., Kang L., Hu L. (2024). Corynoline Protects Chronic Pancreatitis via Binding to PSMA2 and Alleviating Pancreatic Fibrosis. J. Gastroenterol..

[168] Yong Q., Huang C., Chen B., An J., Zheng Y., Zhao L., Peng C., Liu F. (2024). Gentiopicroside Improves NASH and Liver Fibrosis by Suppressing TLR4 and NLRP3 Signaling Pathways. Biomed. Pharmacother..

[169] Song J., He G.-N., Dai L. (2023). A Comprehensive Review on Celastrol, Triptolide and Triptonide: Insights on Their Pharmacological Activity, Toxicity, Combination Therapy, New Dosage Form and Novel Drug Delivery Routes. Biomed. Pharmacother..

[170] Wang K., Zhu K., Zhu Z., Shao F., Qian R., Wang C., Dong H., Li Y., Gao Z., Zhao J. (2023). Triptolide with Hepatotoxicity and Nephrotoxicity Used in Local Delivery Treatment of Myocardial Infarction by Thermosensitive Hydrogel. J. Nanobiotechnology.

[171] Wang X., Wang Q., Li W., Zhang Q., Jiang Y., Guo D., Sun X., Lu W., Li C., Wang Y. (2020). TFEB-NF-κB Inflammatory Signaling Axis: A Novel Therapeutic Pathway of Dihydrotanshinone I in Doxorubicin-Induced Cardiotoxicity. J. Exp. Clin. Cancer Res. CR.

[172] Fang R., Zhou R., Ju D., Li M., Wang H., Pan L., Wang X., Han M., Yu Y. (2024). Zhen-Wu-Tang Protects against Myocardial Fibrosis by Inhibiting M1 Macrophage Polarization via the TLR4/NF-κB Pathway. Phytomedicine.

[173] Yang M., Tan Y.-Q., Zhang J.-Y., Zhang Y., Su L.-Q., Hu Z.-X., Hu S.-Y. (2024). Shenfu Injection Regulates Macrophage Polarization via TLR4/NF-κB Pathway to Reduce Inflammation in Chronic Heart Failure. Zhongguo Zhong Yao Za Zhi.

[174] Ni S., He X., Ouyang X., Zhang X., Li J., Chen X., Liu D., Sun S., Li Z., Li S. (2024). Effects and Mechanisms of Xinyang Tablets Improving Uremic Cardiomyopathy Mice by Inhibiting Osteopontin-Mediated Cardiac Fibrosis Signaling. China J. Tradit. Chin. Med. Pharm..

[175] Shi L., Deng J., Yin E., Chen X., Du X. (2023). Effect of Fangji Fulingtang on Macrophage Polarization and Oxidative Stress in Mouse Model of Myocardial Fibrosis. Chin. J. Exp. Tradit. Med. Formulae.

[176] Anwaier G., Xie T.-T., Pan C.-S., Li A.-Q., Yan L., Wang D., Chen F.-K., Weng D.-Z., Sun K., Chang X. (2022). QiShenYiQi Pill Ameliorates Cardiac Fibrosis After Pressure Overload-Induced Cardiac Hypertrophy by Regulating FHL2 and the Macrophage RP S19/TGF-Β1 Signaling Pathway. Front. Pharmacol..

[177] Huang Y., Zhang K., Liu M., Su J., Qin X., Wang X., Zhang J., Li S., Fan G. (2021). An Herbal Preparation Ameliorates Heart Failure with Preserved Ejection Fraction by Alleviating Microvascular Endothelial Inflammation and Activating NO-cGMP-PKG Pathway. Phytomedicine.

[178] Liu B., He Y., Lu R., Zhou J., Bai L., Zhang P., Ye S., Wu J., Liang C., Zhou Y. (2018). Zhen-Wu-Tang Protects against Podocyte Injury in Rats with IgA Nephropathy via PPARγ/NF-κB Pathway. Biomed. Pharmacother..

[179] Han Y., Huang L., Zhong G., Chang X., Zhu Q., Xu M., Mingtai C., Men L., Wang L. (2022). Evaluation of the Safety and Efficacy of Zhenwu Decoction as Adjuvant Therapy for the Treatment of Heart Failure with Reduced Ejection Fraction: A Protocol for Systematic Review and Meta-Analysis. Medicine.

[180] Tang Q., Wang Y., Li K. (2018). Zhenwu Decoction for Chronic Heart Failure: Protocol for a Systematic Review and Meta-Analysis. Medicine.

[181] Liu B., Cao Y., Wang D., Zhou Y., Zhang P., Wu J., Chen J., Qiu J., Zhou J. (2021). Zhen-Wu-Tang Induced Mitophagy to Protect Mitochondrial Function in Chronic Glomerulonephritis via PI3K/AKT/mTOR and AMPK Pathways. Front. Pharmacol..

[182] Mok H.L., Cheng K.W., Xu Y., Huang C., Lyu C., Xu J., Hu D., Zhu L., Lin C., Tan H.-Y. (2024). Modified Zhenwu Decoction Suppresses Chronic Colitis via Targeting Macrophage CCR2/Fyn/P38 MAPK Signaling Axis. Phytomedicine.

[183] Wang J., Wang X., Wan W., Guo Y., Cui Y., Liu W., Guo F. (2021). Effects of Shenfu Injection on Myocardial Adenosine Receptors in Rats with Myocardial Ischemia-Reperfusion Postconditioning. Hum. Exp. Toxicol..

[184] Liao J., Qin C., Wang Z., Gao L., Zhang S., Feng Y., Liu J., Tao L. (2024). Effect of Shenfu Injection in Patients with Septic Shock: A Systemic Review and Meta-Analysis for Randomized Clinical Trials. J. Ethnopharmacol..

[185] Tao L., Mo Z., Li Z., Li S., Luo Z., Li D., Wang D., Zhu W., Ding B. (2023). Efficacy and Safety of Shenfu Injection on Acute Heart Failure: A Systematic Review and Meta-Analysis. Phytomedicine.

[186] Wu Y., Li S., Li Z., Mo Z., Luo Z., Li D., Wang D., Zhu W., Ding B. (2022). Efficacy and Safety of Shenfu Injection for the Treatment of Post-Acute Myocardial Infarction Heart Failure: A Systematic Review and Meta-Analysis. Front. Pharmacol..

[187] Xu F.-F., Xie X.-F., Hu H.-Y., Tong R.-S., Peng C. (2024). Shenfu Injection: A Review of Pharmacological Effects on Cardiovascular Diseases. Front. Pharmacol..

[188] Wu H., Wu W., Li R., Chen Y., Wang S. (2011). Effects of Xinyang Tablet on Brain Natriuretic Peptide, Ultrasensitive C-Reactive Protein and Cardiac Function in Patients with Acute Decompensated Heart Failure. Tradit. Chin. Drug Res. Clin. Pharmacol..

[189] Salloum F.N., Chau V.Q. (2019). Osteopontin in HFpEF: More Than Just a Remodeling-Specific Biomarker. J. Am. Coll. Cardiol..

[190] Wu G., Yao Z., Zhang X., Li G. (2022). Effect of Fangji Fuling Tang Adjuvant Therapy on Orphan Nuclear Receptors and Ventricular Remodeling in Peripheral Blood Mononuclear Cells from Patients with Refractory Heart Failure. Clin. J. Chin. Med..

[191] Wang M., Shan Y., Wu C., Cao P., Sun W., Han J., Shen L., Chen J., Yu P., Chen X. (2020). Efficacy and Safety of Qishen Yiqi Dripping Pill for Heart Failure with Preserved Ejection Fraction: A Systematic Review and Meta-Analysis. Front. Pharmacol..

[192] Zhou Z., Wang S., Fan Z., Zhang Z., Zhang X., Zhao Z., Wang X., Mao J. (2025). Effect of Qishen Yiqi Dripping Pills on the Classification of Ejection Fraction in Patients with Ischaemic Heart Failure: A Prospective Cohort Study. Phytomedicine.

[193] Kittleson M.M., Panjrath G.S., Amancherla K., Davis L.L., Deswal A., Dixon D.L., Januzzi J.L., Yancy C.W. (2023). 2023 ACC Expert Consensus Decision Pathway on Management of Heart Failure with Preserved Ejection Fraction: A Report of the American College of Cardiology Solution Set Oversight Committee. J. Am. Coll. Cardiol..

[194] Usman M.S., Bhatt D.L., Hameed I., Anker S.D., Cheng A.Y.Y., Hernandez A.F., Jones W.S., Khan M.S., Petrie M.C., Udell J.A. (2024). Effect of SGLT2 Inhibitors on Heart Failure Outcomes and Cardiovascular Death across the Cardiometabolic Disease Spectrum: A Systematic Review and Meta-Analysis. Lancet Diabetes Endocrinol..

[195] Chen P., Ning X., Li W., Pan Y., Wang L., Li H., Fan X., Zhang J., Luo T., Wu Y. (2022). Fabrication of Tβ4-Exosome-Releasing Artificial Stem Cells for Myocardial Infarction Therapy by Improving Coronary Collateralization. Bioact. Mater..

[196] Rai A., Claridge B., Lozano J., Greening D.W. (2024). The Discovery of Extracellular Vesicles and Their Emergence as a Next-Generation Therapy. Circ. Res..

[197] Izzo A.A., Borrelli F., Capasso R. (2002). Herbal Medicine: The Dangers of Drug Interaction. Trends Pharmacol. Sci..

